# Nature-Based Solution to Eliminate Cyanotoxins in Water Using Biologically Enhanced Biochar

**DOI:** 10.1021/acs.est.3c05298

**Published:** 2023-10-19

**Authors:** Jane Moore, Anjali Jayakumar, Sylvia Soldatou, Ondřej Mašek, Linda A Lawton, Christine Edwards

**Affiliations:** † CyanoSol, School of Pharmacy and Life Sciences, Robert Gordon University, Aberdeen AB10 7AQ, U.K.; ‡ School of Engineering, Newcastle University, Newcastle Upon Tyne NE1 7RU, U.K.; § UK Biochar Research Centre, School of GeoSciences, University of Edinburgh, Edinburgh EH9 3JW, U.K.; ∥ Marine Biodiscovery Centre, Department of Chemistry, University of Aberdeen, Aberdeen AB25 1HG, U.K.

**Keywords:** biological water treatment, eutrophication, waste valorization, microcystins, biodegradation, microbiome

## Abstract

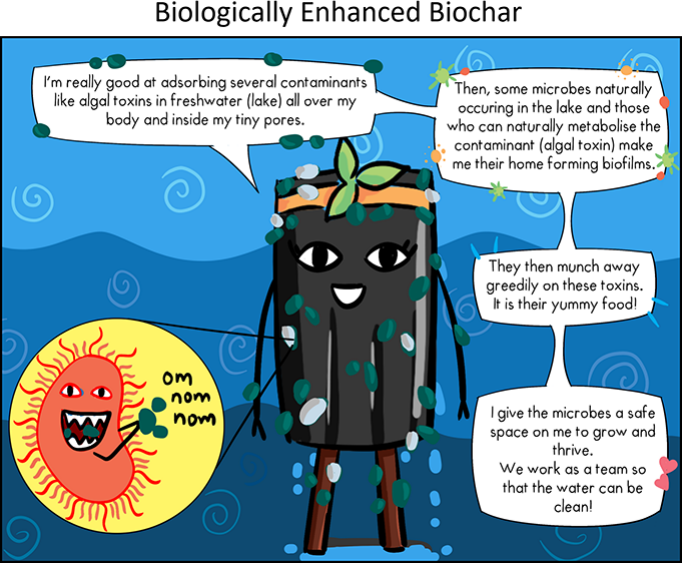

Climate change and high eutrophication levels of freshwater sources are increasing the occurrence and intensity of toxic cyanobacterial blooms in drinking water supplies. Conventional water treatment struggles to eliminate cyanobacteria/cyanotoxins, and expensive tertiary treatments are needed. To address this, we have designed a sustainable, nature-based solution using biochar derived from waste coconut shells. This biochar provides a low-cost porous support for immobilizing microbial communities, forming biologically enhanced biochar (BEB). Highly toxic microcystin-LR (MC-LR) was used to influence microbial colonization of the biochar by the natural lake-water microbiome. Over 11 months, BEBs were exposed to microcystins, cyanobacterial extracts, and live cyanobacterial cells, always resulting in rapid elimination of toxins and even a 1.6–1.9 log reduction in cyanobacterial cell numbers. After 48 h of incubation with our BEBs, the MC-LR concentrations dropped below the detection limit of 0.1 ng/mL. The accelerated degradation of cyanotoxins was attributed to enhanced species diversity and microcystin-degrading microbes colonizing the biochar. To ensure scalability, we evaluated BEBs produced through batch-scale and continuous-scale pyrolysis, while also guaranteeing safety by maintaining toxic impurities in biochar within acceptable limits and monitoring degradation byproducts. This study serves as a proof-of-concept for a sustainable, scalable, and safe nature-based solution for combating toxic algal blooms.

## Abstract 2

Biologically enhanced biochar, a sustainable and cost-effective solution for the removal of microcystins from contaminated drinking water.

## 1 Introduction

Decentralizing drinking water treatment using locally available resources is essential to achieving the UN Sustainable Development Goal (SDG) 6: clean water and sanitation for all.^1^ Conventional water treatment plants require substantial investment and heavily engineered distribution systems, while often not achieving the removal of highly toxic pollutants.^2^ Climate change and nutrient enrichment of drinking water sources are adding to water stress, particularly through the widespread occurrence of cyanobacterial blooms (blue-green algae), which produce potent cyanotoxins and increase water treatment costs.^3−5^ Ingestion of cyanotoxins, particularly microcystins, results in hepatoxicity and cell damage, resulting in fatalities, such as in Caruaru, Brazil, with over 60 reported fatalities in 1996.^6−10^ The fatalities reported in Brazil were attributed to the use of microcystin-contaminated water for dialysis.^6^ There are also growing concerns that microcystins may be responsible for rising cases of chronic kidney disease.^6,7,11,12^

Typical water treatment processes may include dissolved air floatation, coagulation, and flocculation, with cyanobacterial cell removal efficiencies of 71 to 99% and 30 to >90%, respectively. These techniques rely on the removal of whole cells and are not as effective against the free microcystins released upon cell lysis.^13^ Sand filtration is another commonly used water treatment technique, which can effectively remove up to 94% of microcystins under optimal conditions.^10,13^ However, reductions in temperature to 0–10 °C have been found to greatly reduce efficiency, with only 43% microcystin removal reported. Sand filtration also relies on long water residing times of 2–6 months for optimal microcystin removal, which is not always feasible; therefore, rapid sand filtration methods with reduced residing times are often employed. These methods are less efficient, particularly on exposure to elevated microcystin concentrations, above 0.6 μg/L, with only 10% microcystin removal reported in some cases.^13^

In conjunction with the aforementioned water treatment methods, additional disinfection processes such as chlorination, ozone, or activated carbon may be employed. Chlorination has been shown to remove up to 99% of microcystin in lab-scale studies, however, the formation of undesirable toxic byproducts is problematic.^14,15^ Ozone treatment of drinking water can effectively remove microcystins; however, the process requires close monitoring as the amount of ozone required to achieve this varies depending on the water quality.^10,16^ Combination of these methods with UV irradiation has been shown to improve their efficacy.^15,17^

These water treatment systems are highly engineered, requiring extensive infrastructure and monitoring for effective water treatment. Therefore, novel, innovative, simple, cost-effective, and sustainable solutions are required. In this proof-of-concept study, we demonstrate the potential of biologically enhanced biochar (BEB) as a sustainable and economical water treatment solution.

The adsorptive capabilities of activated carbon, powdered or granular (PAC or GAC), find extensive application in tertiary water treatment scenarios.^10,18^ These systems have demonstrated effective microcystin removal at both lab scale and within water treatment facilities, with complete microcystin removal and 49–87% microcystins (maintaining a final drinking water concentration of 0.05–0.18 μg/L microcystins) removal, respectively.^19,20^ To improve water treatment efficiency, the absorptive capabilities of activated carbon have been combined with the biological degradation capabilities of microorganisms in biologically activated carbon (BAC). These systems have been reported to remove 20 μg/L microcystins from contaminated water supplies after 16 days of incubation.^21^

The similarities in the mechanisms of BAC operation to our proposed BEB technology prompted us to do a detailed and direct comparison of their cost-effectiveness, environmental performance, contaminant removal efficiencies, and end-of-life scenarios in our review paper.^18^ While the BAC process has been shown to remove several organic/inorganic contaminants, including microcystins, via adsorptive and biodegradation mechanisms, our extensive literature survey showed that BEBs have the potential to be more cost-effective while having lower environmental footprints and still being highly effective in removing contaminants when engineered correctly, especially useful in low- and middle-income countries. More details on mechanisms and cost-environmental analysis are available in our review paper.^18^ This review also forms the basis of the experimental methodology adopted in this study.

The adsorptive capabilities of biochar have been demonstrated for environmental applications, including remediation of contaminated soil and water.^10,18,22−24^ However, here we utilize this carbon-based biochar matrix for microbial colonization so that the combined degradative capabilities of the natural freshwater microbiome and adsorptive capabilities of the biochar can be utilized for the complete removal of toxic microcystins.

**Figure 1:**
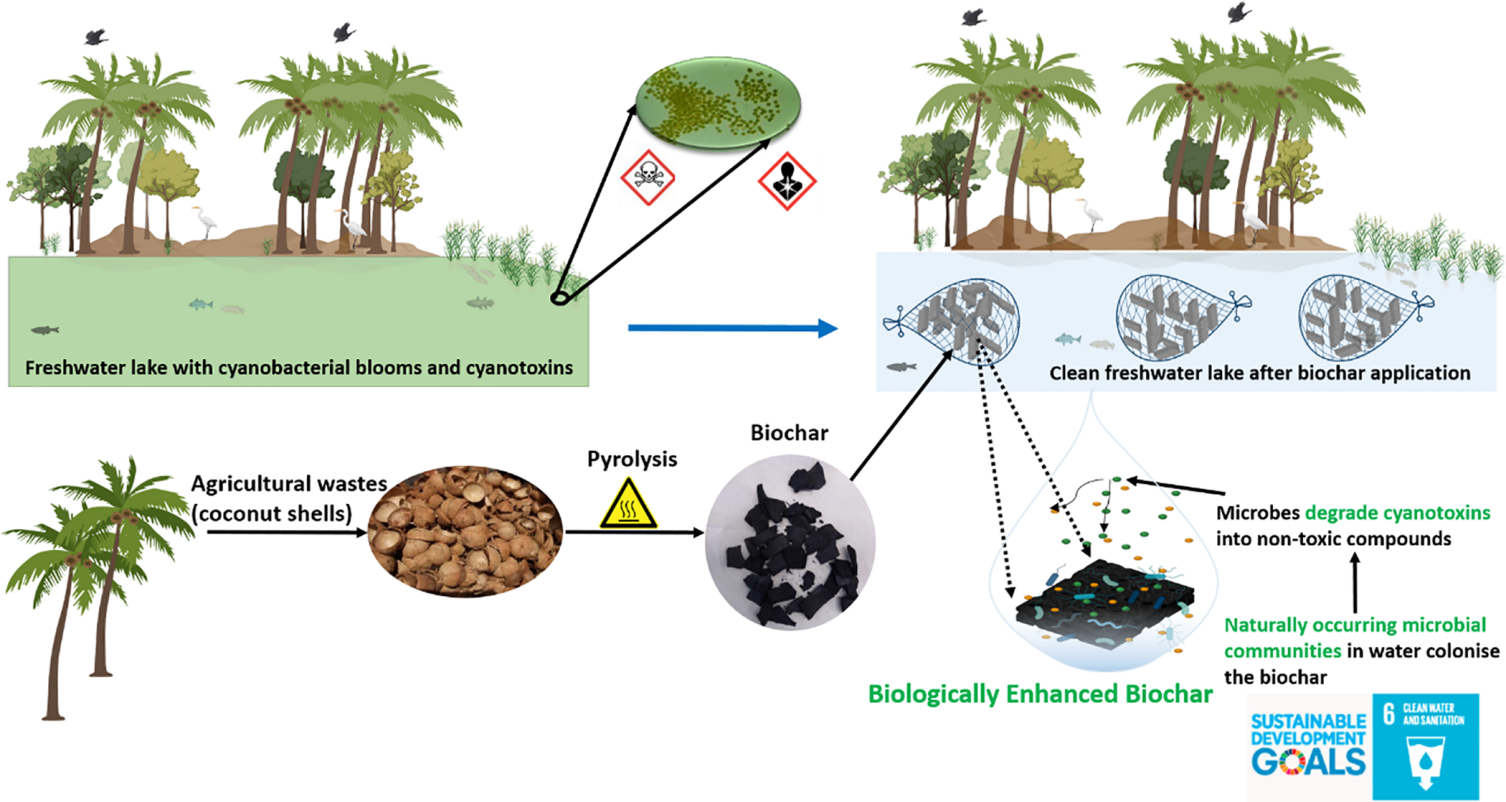
Nature-based solution for water treatment using BEB. Schematic of biochar production and proposed application of the technology.

Previous work has found that the natural freshwater microbial consortia contain active biodegraders that eliminate cyanotoxins with degradation half-lives of 4–18 days.^25−27^ Several freshwater bacterial species have been identified that are capable of degrading microcystins, including *Sphingomonas* sp., *Sphingopyxis* sp., *Novosphigobium* sp., *Stenotrophomonas* sp., and *Bacillus* sp. Specifically, *Sphigomonas* sp. ACM-3962 was the first organism found to be capable of utilizing microcystins as a sole carbon and nitrogen source, utilizing the *mlr* gene cluster for microcystin degradation.^26^ This study aims to harness and stimulate this microbial capability by naturally immobilizing freshwater microorganisms on biochar, a carbon-rich product of the thermochemical conversion of biomass, to provide a scalable water treatment system that can be used at all scales from rural wells through to municipal water treatment facilities in diverse global and socioeconomic settings, Figure 1.

## 2 Materials and Methods

### 2.1 Biochar Production

Batch and continuous flow pyrolysis units (also referred to as Stage 2 pyrolysis units) were used for biochar production to allow us to compare the quality and functionality of biochar produced using small and larger-scale production units.

Coconut shells were procured from commercial suppliers in India (Annapoorneswari Tech, India). The coconut shells were cleaned, dried, and crushed to an average size of 2–3 cm, then pyrolyzed using a vertical batch reactor or continuous flow pyrolysis unit (also referred to as Stage 2 pyrolysis unit) to produce coconut shell biochar at the UK Biochar Research Centre as per the protocols previously described.^28−30^

During the pyrolysis process, the coconut shells (70–80 g) were first purged with N_2_ for 10–15 min to remove any residual oxygen that could hinder the pyrolysis process. Coconut shells were then pyrolyzed in a nitrogen atmosphere in a pyrolysis glass tube reactor (borosilicate glass for 450 and 550 °C, and quartz for 700 °C), and for the continuous flow pyrolysis unit at a flow rate of 1 L min^–1^ with average residence times of approximately 40 min. Three different sets of conditions are described in the Supporting Information Table S1 where the temperature, 450, 550, and 700 °C refers to the highest treatment temperature (HTT).

The pyrolysis process generates three products: solid biochar; volatiles, which can be condensed using several hot and cold traps to yield condensable liquids; and finally, syngas. For our application, we utilized the solid biochar produced at 450, 550, and 700 °C. The HTT used for biochar production is known to impact the properties of the biochar such as its composition, specific surface area, structure, pore-size distribution, surface functional groups, and pH.^30^ By using coconut biochar produced at 3 different HTTs (450, 550, and 700 °C) the effect of the different biochar properties on microbial colonization and subsequent microcystin adsorption and biodegradation could be assessed.

### 2.2 Coconut Shell Biochar Characterization

Coconut shell biochar was crushed and sieved to <1 mm for all characterizations.

Coconut shell biochar yields were calculated on a dry basis (d.b.) as a percentage of total dry weight, without accounting for moisture, denoted as wt % d.b. This was done by measuring the weight difference of the feedstock and the produced biochar before and after pyrolysis.^30^ Biochar yield is calculated using eq A1 provided below. All values are on a moisture-free, dry basis.
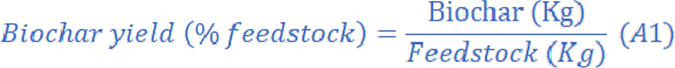
A1

Volatile matter (VM), fixed carbon (FC), and ash content were determined by thermogravimetric analysis using a TGA/DSC 1; Mettler-Toledo, Leicester, UK, by the standard methods used for biochar (in quadruplicates).^30,31^ The moisture content was evaluated after the samples were first heated at 105 °C for 10 min in a N_2_ atmosphere; then the temperature was raised to 900 °C at 25 °C min^–1^ and was kept there for 10 min to account for VM. Following this hold time, the samples were finally combusted with air at 900 °C for 15 min to determine the ash content of each sample. The percentage of VM, FC, and ash content could then be calculated on a dry basis (d.b.) as a percentage of total dry weight, without accounting for moisture, denoted as wt % d.b.^30,31^

C, H, N, and O compositions of biochar were determined using wt % d.b. by ultimate or elemental analysis using flash combustion on a Thermo Fisher Scientific Flash SMART instrument. All analyses were performed in triplicate for each test sample.

For biochar pH and electrical conductivity (EC) measurements, the standard protocol for biochar samples was followed using a HACH Multi-parameter meter.^32^ All analyses were performed in duplicate for each test sample. In brief, 2 g of biochar was dispersed in 40 mL of deionized water (DW) and then mechanically shaken for 1 h at 25 °C on an orbital shaker. This suspension was left undisturbed for 30 min, and the supernatant was used for pH and EC measurements.

Raman spectroscopy was performed on biochar samples by using a Renishaw inVia Raman microscope with a laser excitation wavelength of 514 nm. A Smiths Illuminat IR module was mounted on the same microscope for recording the FTIR spectra of the biochar samples.

Surface area measurements of biochar samples were performed in duplicate for each test sample. This analysis was performed using N_2_ physisorption at 77K in a Micromeritics Gemini 2380 in the pressure range 0.01–0.99 after degassing at 300 °C for 3 h.

The surface area of biochar samples was determined from N_2_ adsorption isotherms in the pressure range of 0.05–0.3 using a pore nonspecific method proposed by Brunauer–Emmett–Teller (BET), currently recommended by the European Biochar Certificate (EBC) guidelines.^31^

Biochar samples were analyzed for toxic US 16 EPA PAHs or polycyclic aromatic compounds by MAS GmbH, an accredited laboratory for testing biochar samples, as recommended in the EBC guidelines.^31^ The protocol involved a 36-h Soxhlet extraction of finely crushed biochar samples (<1 mm) followed by a gas chromatography–mass spectrometry (GC-MS) analysis to quantify US 16 EPA PAHs.

### 2.3 Water Collection and Analysis

Surface water samples from Rescobie Loch, Angus, Scotland, 56°39′19’N 2°47′47’W, were collected in 1 L sterilized glass bottles and transferred to the laboratory, where the biodegradation experiments started on the same day. In addition, three samples were collected in 500 mL sterilized glass bottles for water analysis purposes, conducted by James Hutton Limited (Aberdeen, UK, https://www.huttonltd.com/).

At the James Hutton Institute, the Loch water samples were analyzed for total organic carbon (TOC), total nitrogen (TN), chemical oxygen demand (COD), biological oxygen demand (BOD), total oxidizable nitrogen (TON), and dissolved organic carbon (DOC), Supporting Information Table S2.^33^

### 2.4 BEB Colonization and Challenge Assays

For each pyrolysis temperature under which coconut shell biochar was produced (450, 550, and 700 °C), samples were prepared by aseptically adding 100 mL of freshly collected Rescobie Loch water to 250 mL sterile Erlenmeyer flasks closed with a cotton wool bung, Supporting Information Figure S1.

The biochar pellets (weight ranging from 0.6 to 1 g) were washed twice with sterile DW, provided by a Milli-Q system (Millipore, Watford, UK). As required for each test flask, 5–6 biochar pellets were added aseptically to each flask containing 100 mL of Rescobie Loch water. Where required, filter-sterilized microcystin-LR (MC-LR), as per Enzo Life Sciences, was then aseptically added to each flask, resulting in a final concentration of 5 μg/mL MC-LR.

Each sample set was prepared in triplicate for the analysis of the microcystin-degrading capabilities of microorganisms immobilized on the surface of coconut biochar produced at 3 different pyrolysis temperatures (450, 550, and 700 °C). The following test/control samples were included: (1) control (A), containing coconut biochar, 5 μg/mL MC-LR, and sterilized Rescobie Loch water; therefore, no live microcystin-degrading microorganisms; (2) control (B), containing no biochar, 5 μg/mL MC-LR, and nonsterile Rescobie Loch water; therefore, live microorganisms with the potential to degrade microcystins; (3) control (C), containing coconut biochar, no MC-LR, and nonsterile Rescobie Loch water; therefore, live microorganisms with the potential to degrade microcystins; (4) test sample (S), containing coconut biochar, 5 μg/mL MC-LR, and nonsterile Rescobie Loch water; therefore, live microorganisms with the potential to degrade microcystins and biochar.

All samples were incubated at 25 °C under static conditions for a maximum duration of 24 days or until microcystin concentrations in the test samples was below the detectable levels (0.1 ng/mL). Aliquots of 1 mL were aseptically removed during the assay at 12–72 h time intervals as required. The samples were stored at −20 °C for the UPLC-PDA-MS/MS analysis.

To demonstrate that the microcystin-degrading microorganisms were immobilized on the surface of the biochar pellets and to assess the capabilities of these organisms to degrade different microcystins, 14 different challenge experiments were performed, (Supporting Information Tables S3 and S4).

Samples were prepared by adding 100 mL of sterile Loch water in 250 mL sterile Erlenmeyer flasks and closed with a cotton wool bung. Filter-sterilized microcystins/cyanobacteria were added to each flask as required, and then the biochar pellets (450, 550, and 700 °C) from the previous challenge assay were aseptically transferred to the corresponding flask, Supporting Information Tables S3 and S4.

The new flasks containing the coconut biochar transferred from the previous assay were incubated as before at 25 °C under static conditions. Aliquots of 1 mL were aseptically removed during the assay at 12–72 h time intervals as required from the sterile controls and test samples. The samples were stored at −20 °C for UPLC-PDA-MS/MS or UPLC-PDA-QTOF-MSE and -MS/MS analysis.

### 2.5 Ultrahigh Performance Liquid Chromatography Coupled to Photodiode Array Detection and Tandem Mass Spectrometry (UPLC-PDA-MS/MS)

Biodegradation of MC-LR was analyzed by UPLC-PDA-MS/MS (Waters, Manchester, UK) as described previously.^34^

Prior to analysis, all samples were centrifuged at 14,000 rpm for 5 min and then diluted 1 in 10 in DW as required.

Chromatographic separation was carried out using a Waters Acquity UPLC BEH C18 column (1.7 μm, 2.1 mm × 50 mm) held at 60 °C. Samples were kept in the sample manager at 10 °C and the injection volume was 5 μL. The mobile phase consisted of (A) water + 0.025% formic acid and (B) acetonitrile +0.025% formic acid at a flow rate of 0.6 mL/min. The gradient consisted of 2% B initial condition increasing to 25% B at 0.5 min holding until 1.5 min, increasing to 40% B at 3.0 min, rising further to 50% B at 4 min, a quick rise to 95% B and 4.1 min and held until 4.5 min before dropping back to 2% B at 5 min.

LC-MS-grade acetonitrile, methanol, and formic acid were purchased from Sigma-Aldrich (Irvine, UK). DW was provided by a Milli-Q system (Millipore, Watford, UK).

Mass spectrometric detection was performed with a triple quadrupole mass spectrometer (Waters Xevo TQ-XS, Manchester, UK) equipped with an electrospray ionization (ESI) source operating in positive ionization mode. Operational parameters were as follows: 150 °C source temperature, 600 °C desolvation temperature, 600 L/h desolvation gas flow (N_2_), 150 L/h cone gas flow, and 0.15 mL/min collision gas flow (Ar). Capillary voltage was held at 1.0 kV. Quantification was carried out using an external calibration curve based on an 11-point calibration ranging from 0.5 to 500 ng/mL of microcystin. The detection limit was 0.5 ng/mL and the quantification limit was 1.0 ng/mL. Acquisition and processing of MS data were done using MassLynx v 4.2 software (Waters, UK).

MC-LR, as per Enzo Life Sciences, was used for external calibration; an 11-point calibration curve was prepared by serial dilution within the range of 0.5–500 ng/mL.

### 2.6 UPLC–PDA Coupled to Quadrupole Time of Flight Mass Spectrometry (QTOF-MSE, QTOF-MS/MS)

Analysis of the *Microcystis aeruginosa* B2666 cyanotoxins and microcystin biodegradation products and quantification of aeruginosins and cyanopeptolin was carried out using UPLC-PDA-QTOF-MSE and -MS/MS (Waters, UK) equipped with an ESI source. Prior to analysis, all samples were centrifuged at 14,000 rpm for 5 min.

Compound separation was achieved using a C18 BEH column (1.7 μm, 2.1 mm × 100 mm) held at 40 °C. The mobile phase was acetonitrile with 0.1% formic acid (B) and water with 0.1% formic acid (A) at a flow rate of 0.2 mL/min. Gradient elution was as follows: 20% B initial condition rising to 70% B at 9.50 min, increasing further to 100% B at 10 min, holding until 11 min, dropping back to 20% B at 12 min, and holding until 14 min.

The QTOF was operated in positive ESI mode. The operational parameters were the following: 3.0 kV capillary voltage, 40 V cone voltage, 100 °C source temperature, 250 °C desolvation temperature, 150 L/h cone gas flow, and 600 L/h desolvation gas flow. Argon was used as the collision gas. MS/MS consisted of four functions: the first function used a collision energy ramp of 25–45 eV to acquire MSE data; the second and third functions used a collision energy ramp of 25–45 eV for the targeted masses at a scan time of 0.1 s; and the fourth function acquired the lock mass for online mass calibration. Leucine-Enkephalin (*m*/*z* 556.2771 for positive electrospray mode) was infused at a flow rate of 10 μL/min at 10 s intervals as lock mass. Acquisition and processing of MS data were done using MassLynx version 4.2 software (Waters, Manchester, UK).

MC-LR and MC-LA were identified by characteristic low and high energy mass spectra (SI) as the predominant MCs in extracts and cultures of *M. aeruginosa* B2666, as previously reported by Diehnelt et al.^35^ Other major peptides were identified as cyanopeptolin 1020 based on *m*/*z* 1021.5372 ([M + H]^+^) and a putative aeruginosin at *m*/*z* 601.3358 having the intense fragment in the high energy spectrum at *m*/*z* 140.1077 representing the 2-carboxy-6-hydroxy-octahydroindole (Choi) immonium ion, Supporting Information Figures S2–S5.

### 2.7 Cyanobacterial Culture

The cyanobacterium *M. aeruginosa* B2666 was cultured in BG-11 medium at 21 ± 1 °C on a 12/12 h light/dark cycle illuminated by cool white fluorescent lights (correlated color temperature 1400–5000 K) with an average illumination of 10.5 μmol photons m^–2^ s^–1^ without agitation.^36^

### 2.8 *M. aeruginosa* B2666 Cell Enumeration

A Multisizer 3 (Beckman Coulter, USA) was used to enumerate *M. aeruginosa* B2666 cell density to evaluate biovolume and average cell diameter. A 50 μm aperture was used, which allows particle size detection from 1 to 30 μm. Samples were diluted 20 to 50-fold in isoton carrier liquid (Beckman Coulter, USA), depending on the sample density.

### 2.9 Metagenomic Analysis of the BEBs

Metagenomic analysis was used to assess the genomic diversity of the microbial population colonizing the surface of the coconut biochar. A single pellet was removed from each triplicate of the coconut biochar pyrolysis temperature (450, 550, and 700 °C) samples naïve control C (no MC-LR) and test samples at the end of challenge 4 and again from the test samples at the end of challenge 14.

At NCIMB (Aberdeen), DNA was extracted using DNeasy PowerSoil (QIAGEN), using a modified version of “16S Metagenomic Sequencing Library Preparation” (part no. 15044223 Rev. B, Illumina). This procedure was modified with the use of NEBNext Q5 HiFi Mastermix (New England Biolabs, UK) for DNA amplification of the V1 and V2 hypervariable regions of the 16S rRNA gene using primers (27F - 5′ TCGTCGGCAGCGTCAGATGTGTATAAGAGACAGAGAGTTTGATCCTGGCTCAG 3′/338R - 5′ GTCTCGTGGGCTCGGAGATGTGTATAAGAGACAGGCTGCCTCCCGTAGGAG 3′). Amplicons were sequenced on a MiSeq V2 500 cycle flowcell (Illumina), producing 250 base paired-end reads for analysis. The sequence reads were QC’d and analyzed using CLC Genomics workbench version 22.01 and the SILVA database for taxonomic profiling.

### 2.10 Statistical Analysis

Biochar produced in this study from both batch- and continuous-scale pyrolysis units have been already shown to be consistent and reproducible in their properties across time and production scales, with samples also tested for normality.^37^ Statistical analysis on BEBs produced using coconut shell biochar samples from both batch and continuous scale production was performed using one-way ANOVA tests at a statistical significance level of 0.05 using the Python programming language to test the potential difference in the means of the degradation half-lives of each of the BEB test samples for all the transfers and challenge experiments.

## 3 Results and Discussion

**Figure 2:**
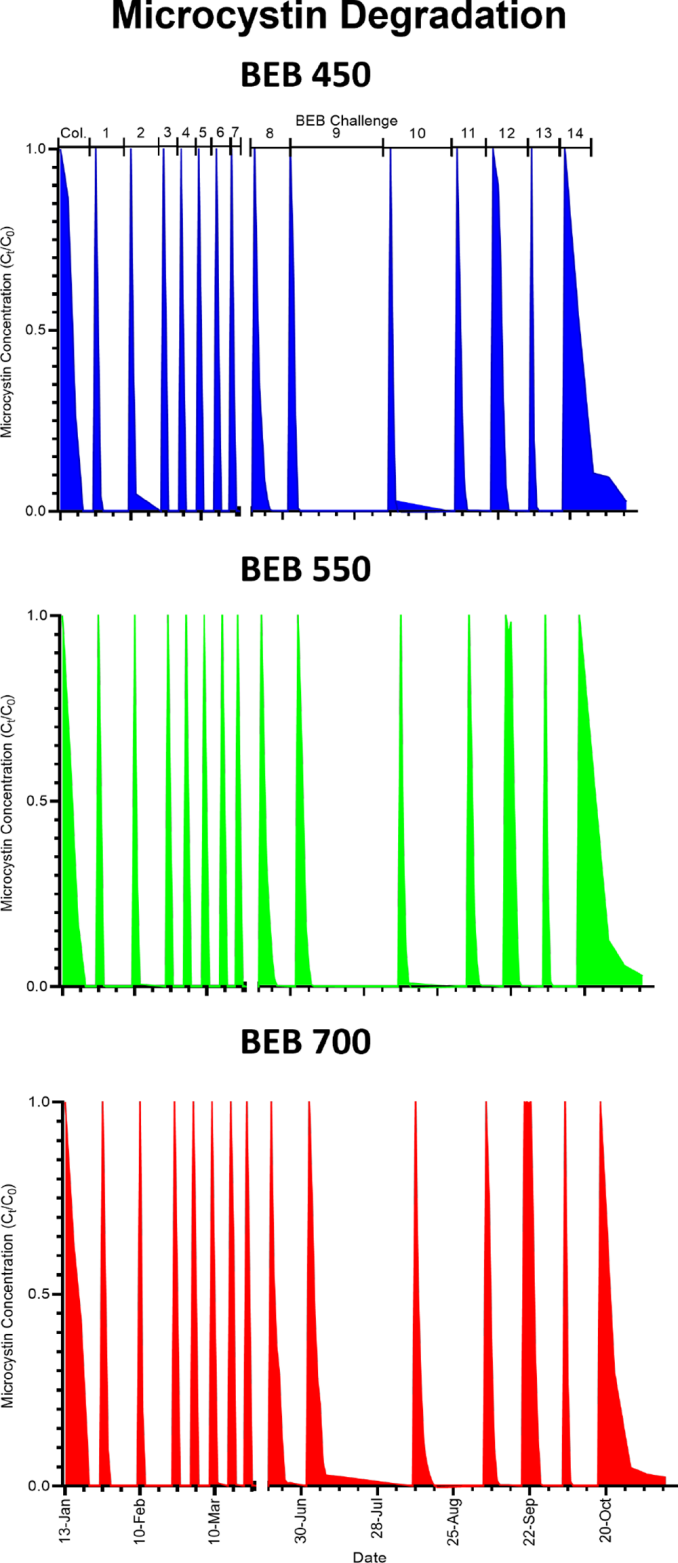
BEB biodegradation capability. Microcystin degradation profile of the biochar after natural microbial colonization (BEB), displaying colonization stage to challenge 14 of BEB450, BEB550, and BEB700.

**Figure 3:**
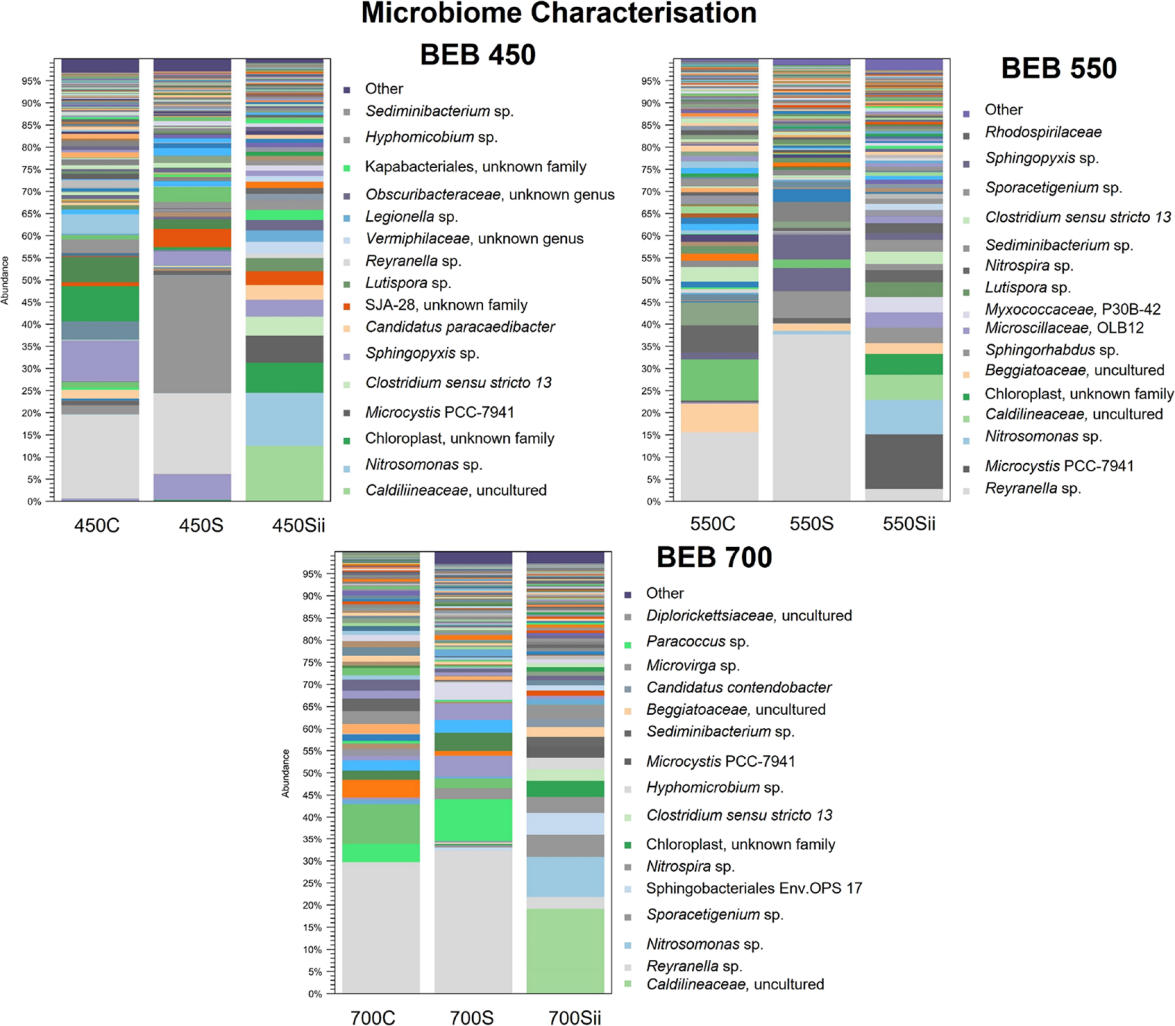
BEB microbiome characterization. Microbiome profile of the BEBs with no microcystin exposure (C), after MC-LR exposure (S), and after exposure to live cyanobacteria (Sii).

We used our biologically enhanced coconut shell biochar continuously for 11 months for 14 different microcystin challenges without biochar replenishment or microbial inoculation, Figure 2. During this time, MC-LR degradation rates were consistent, indicating that the BEB functional lifespan extends beyond the 11 month duration of our investigation. These BEBs were formed by the spontaneous colonization of the biochar by the freshwater microbiome, resulting in the formation of diverse microbial communities colonizing the biochar, Figure 3.

### 3.1 Biochar Production and Characterization

**Figure 4:**
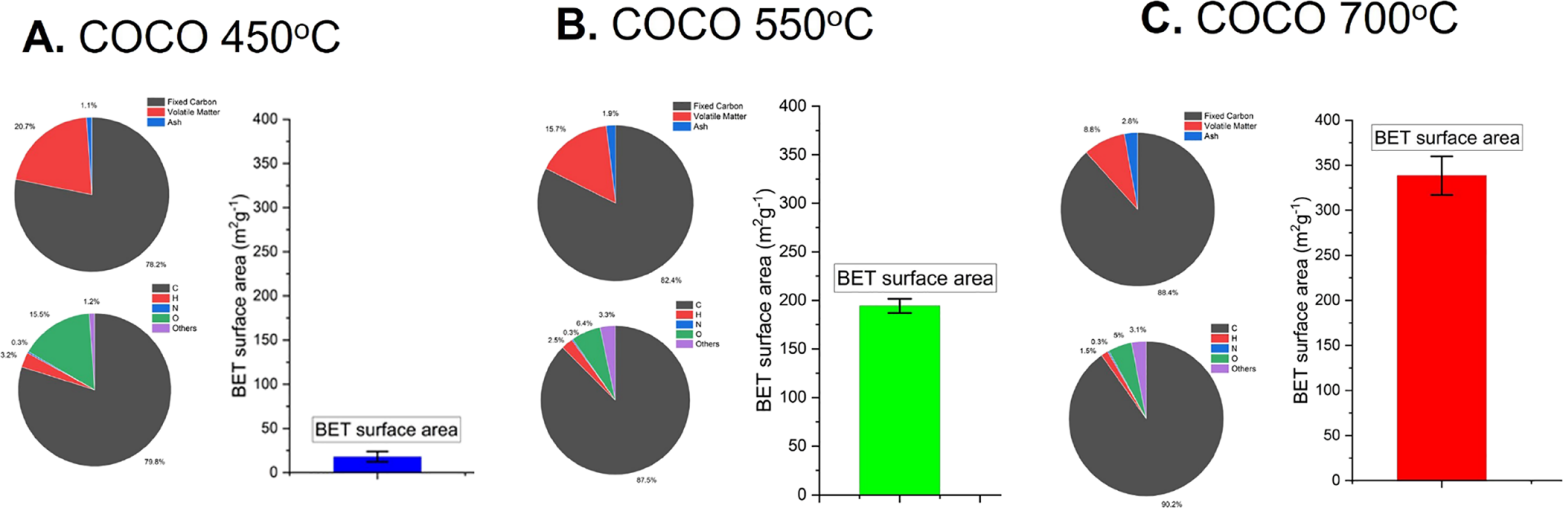
Characterization of coconut shell biochar. Composition from proximate analysis (top pie chart) and elemental analysis (bottom pie chart) and bar plot showing BET surface area for coconut shell biochar produced at three different temperatures: (A) COCO 450, (B) COCO 550, and (C) COCO 700.

Coconut shell biochar was produced using pyrolysis, a thermochemical conversion process under oxygen-deficient conditions. The physiochemical properties of biochar are known to vary depending on the pyrolysis temperature.^38^ Hence, for the optimization of microbial colonization and to evaluate the scalability of the proposed solution, coconut shell biochar was produced using both batch-scale and continuous-scale pyrolyzers under three pyrolysis temperatures (450, 550, and 700 °C), representing typical biochar pyrolysis temperature ranges, Supporting Information Table S1. As biochar production temperatures increased, so did the biochar FC content and specific surface area. On the contrary, the number of oxygen-containing functional groups decreased, Figure 4. To ensure that our biochar was safe to use, the polycyclic aromatic hydrocarbons (PAHs), an undesirable toxic coproduct of biomass pyrolysis, content was assessed. Results showed that the PAH content in all our biochars was below the recommended limits outlined within the International Biochar Initiative standards and European Biochar Certification standards for biochar production and application, Supporting Information Table S5.^28,31,39^

### 3.2 Microbial Colonization of Biochar

To produce the BEBs, the 3 different coconut shell biochars were exposed to fresh lake water containing naturally occurring microorganisms, resulting in spontaneous colonization of the biochar by the freshwater microbiota, Figure 3. Toxin removal capabilities of the combined biochar and freshwater microbiome (BEBs) were then assessed, together with the effects of toxin exposure on the microbial community colonizing the biochar, Figure 2.

We demonstrate that all BEBs, independent of the biochar pyrolysis temperature, could be used to rapidly remove microcystins from contaminated water supplies in 14 different microcystin challenges performed over 11 months, Figure 2.

**Figure 5:**
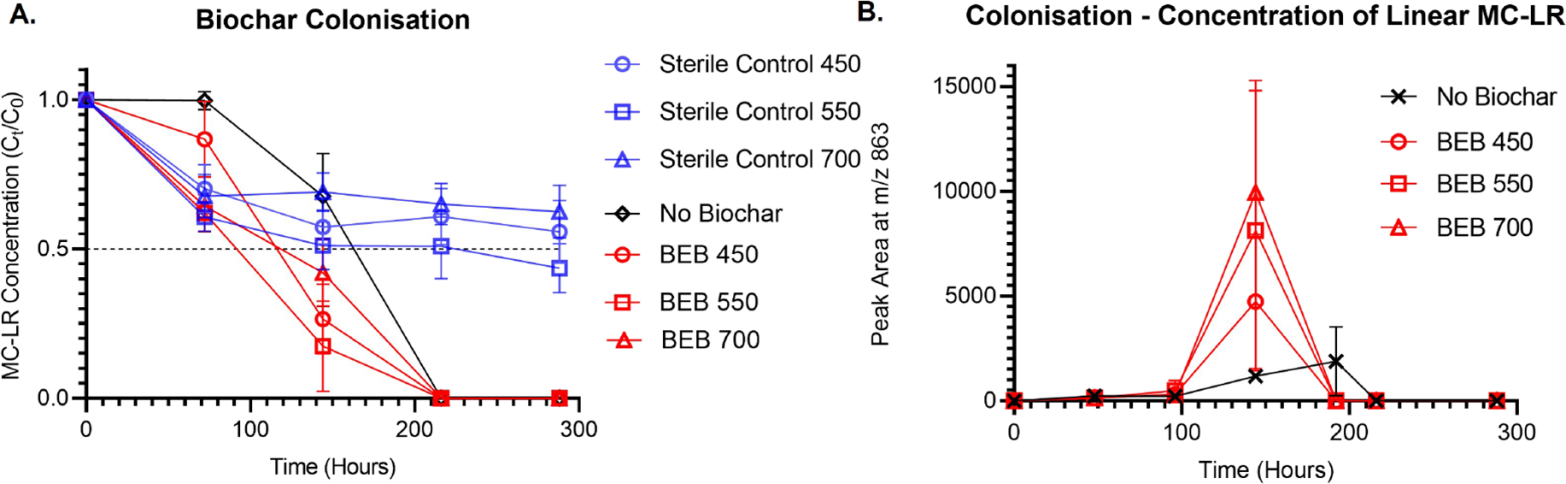
MC-LR degradation during BEB colonization phase. (A) MC-LR degradation profile during the process of spontaneous biochar colonization by the natural freshwater microbiome. (B) Transient detection of a MC-LR degradation product (linear MC-LR) during the biochar colonization phase. Error bars represent the standard deviation *n* = 3.

The first step of this assay, “colonization”, involves the exposure of coconut shell biochar to the naturally occurring freshwater microbiome. During the colonization stage, multiple mechanisms of toxin removal are in play, passive biochar adsorption and active biodegradation (by planktonic microbes found in freshwater and those immobilized as part of the biofilm on the biochar surface).^18^ Therefore, several controls were included to enable us to differentiate between these different mechanisms of toxin removal. Control A, sterile control, allowed us to evaluate the biochar toxin removal capabilities solely based on its adsorptive properties and without the help of microorganisms. Control B, the no biochar control, allowed us to evaluate the toxin removal capabilities of the water-borne planktonic microbes alone. Control C, no toxin control, to assess microbial community structure in the absence of microcystins, Figure 5A and Supporting Information Table S3.

During the first 72 h of colonization, a 25–50% reduction in MC-LR concentration was observed in all biochar-containing samples, Figure 5A. This was observed both in the presence and absence of microorganisms and therefore is attributed to adsorption. It is clear that BEBs are efficiently removing MC-LR; however, in order to obtain safe drinking water, it is imperative to MC-LR by coconut shell biochar, made possible by the rich surface functional groups in biochar (especially for the lower temperature biochar) and larger macro-mesopores capable of adsorbing a large molecule such as MC-LR, Supporting Information Table S5.^10,23^

Following the initial biochar MC-LR adsorption, MC-LR concentrations in the BEB test samples continued to decrease until the MC-LR concentrations were below the quantification limit of 1.0 ng/mL. This reduction in MC-LR concentration observed in the BEB test samples, beyond the adsorption capacity of biochar alone, was attributed to the degradation of MC-LR by naturally occurring microorganisms in Rescobie Loch water, 56°39′19′N 2°47′47′W. This is supported in the literature, where the freshwater microbiome from multiple sources has been shown to degrade microcystins and confirmed by the observation that MC-LR concentration in the no biochar sample was also found to drop below the quantification limit of 1.0 ng/mL, Figure 5A.^25,40,41^

During the biochar colonization phase of this study, the MC-LR concentration dropped below the detection limit in the no biochar control and BEB test samples after 216 h of incubation. However, the MC-LR degradation half-life of the no biochar control sample was 25–42% slower than that of the BEB test samples. This delay in the no biochar control sample 72 h lag phase in the initiation of MC-LR degradation, Figure 5A. Localization of microcystins on the biochar surface may have made it easier for the immobilized microbial community to metabolize and degrade adsorbed toxins due to reduced mass transfer limitations.^10^

It is important to note that MC-LR degradation rates can differ between freshwater samples due to variations in the freshwater microbiome.^25,40^ However, on this occasion, the MC-LR degradation rates observed during the colonization phase of this study are comparable to those previously observed by Edwards et al., during the analysis of freshwater microbiome microcystin degradation capabilities at the same site (Loch Rescobie).^25^

During the colonization stage, and all subsequent BEB challenge assays (discussed in Sections 3.3–3.5), all BEB test samples (BEB 700, BEB 550, and BEB 450, where the numerical values refer to the coconut shell biochar pyrolysis temperature) displayed similar MC-LR degradation rates, indicating that variations in the physiochemical properties of coconut shell biochar (COCO 450, COCO 550, and COCO 700) do not significantly change the cyanotoxin biodegradation capabilities of BEBs, Figure 5A. This is evidenced by the one-way ANOVA tests showing no significant differences between the average degradation half-lives of BEB 450, BEB 550, and BEB 700 across all the colonization, except for challenges 6 and 7, where BEB 700 MC-LR degradation half-lives were ca. 2 h longer, Supporting Information Figures S6–S8.

Similar results and trends were observed on repetition of this assay using coconut shell biochar produced at a scaled-up continuous biochar production facility, Supporting Information Figures S9 and S10, demonstrating the reproducibility and scalability of this technology.

It is clear that BEBs are efficiently removing MC-LR; however, in order to obtain safe drinking water, it is imperative to ensure that no toxic MC-LR breakdown products remain in the water. Analysis was undertaken to look for MC-LR degradation products, Figure 5B and Supporting Information Figure S11. During the colonization stage, a transient presence of linearized MC-LR was detected in all BEBs and No Biochar samples. This is a microcystin breakdown product observed during microcystin degradation via the *mlr* gene cluster, indicating that the freshwater microbiome contains microorganisms that utilize this system for biodegradation.^21^ After 216 h of incubation, no further MC-LR degradation products could be detected, thus indicating this technology has the potential to be safely used for toxic microcystin removal from drinking water. It is noted that, on analysis of water samples from subsequent MC-LR challenge assays, linearized MC-LR degradation products could not be detected, suggesting very rapid and complete degradation of microcystins.

### 3.3 Challenging BEBs with MC-LR

**Figure 6:**
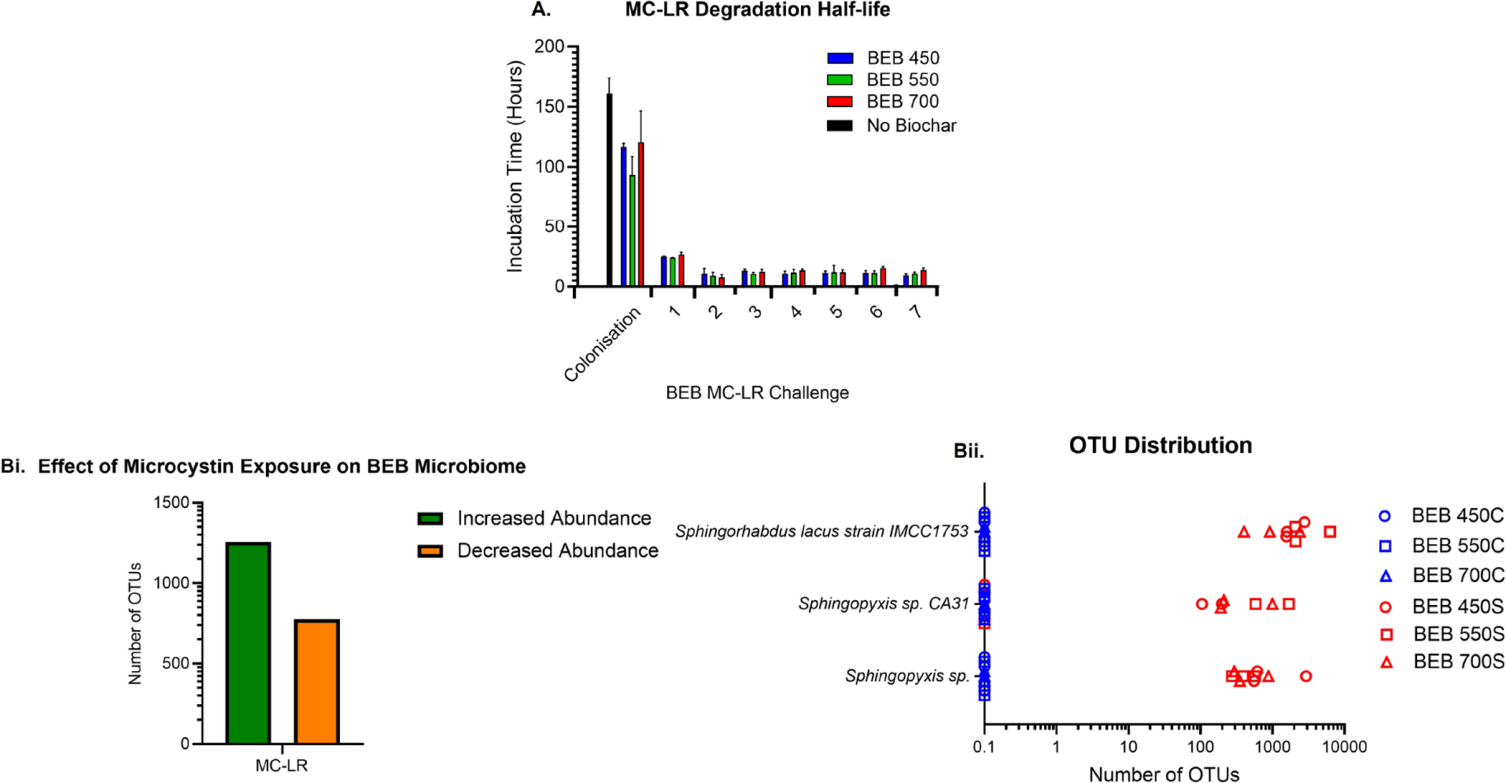
Rapid and efficient MC-LR degradation using BEBs. (A) Microcystin biodegradation half-life, in the presence of BEB, from colonization phase to MC-LR challenge 7. (Bi) Differential abundance analysis displays the number of operational taxonomic units (OTUs) that are at-least 100-fold changed in abundance on the surface of the BEB MC-LR (S) exposed samples compared to the control samples (C). (Bii) Distribution of some of the most abundant OTUs identified on the surface of the different biochar. Error bars represent the standard deviation *n* = 3.

To demonstrate that microorganisms colonizing the biochar surface are responsible for MC-LR degradation, the same BEBs were aseptically transferred into fresh flasks containing sterilized lake water artificially contaminated with MC-LR (5 μg/mL) (challenge 1). MC-LR concentrations in the BEB-containing flasks were monitored until MC-LR concentrations were below detectable levels of 0.1 ng/mL. During challenge 1, not only was the ability to degrade MC-LR retained but in fact enhanced, with ca. 10-fold decrease in the time required to degrade 50% of the MC-LR, Figure 6A. This confirmed that the microorganisms colonizing the biochar were responsible for the degradation of MC-LR, and that, in comparison with the free-living planktonic cells, the colonization of biochar by naturally occurring freshwater microbiome dramatically enhanced the biodegradation capabilities of the freshwater microbiome.

The same BEBs were then aseptically transferred into fresh flasks containing sterilized lake water artificially contaminated with MC-LR for a further 6 challenges (challenge 2–7), over 3 months, to demonstrate the long-term efficacy of BEBs for toxin removal, Figure 6A and Supporting Information Table S3. The increased rate of MC-LR degradation was retained across all 7 MC-LR challenge assays, with a degradation half-life of 13.45 ± 5.22 h, indicating that this water purification system has the potential to be efficient and long-lasting, thus offering a viable practical solution for drinking water treatment. Again, the rate of MC-LR degradation was comparable for all 3 BEB 450, 550, and 700, suggesting we have created a robust system for MC-LR removal from drinking water, Figure 6A.

In comparison with other water treatment processes such as sand filtration and BAC, the MC-LR degradation times are considerably shorter.^21^ There have been several studies focusing on the degradation of MC-LR by bacterial isolates. Only a few of these assess the ability of freshwater microbial communities to eliminate cyanotoxins; however, the rates of MC-LR degradation were 10-fold slower than the 13.45 h MC-LR degradation half-life achieved here with the use of BEBs.^25,40^

To gain insight into the identity of the MC-LR-degrading microorganisms colonizing the biochar, 16S metagenomic analysis was conducted at the end of challenge 4. The BEB microbial community of MC-LR-exposed test samples and naive control C (no MC-LR) samples were compared, Figure 6B,C. Both BEB control and test samples were found to support diverse microbial communities. Exposure to MC-LR was found to alter the microbiome. On comparison of the abundance of individual operational taxonomic units (OTUs) ca. 1000 OTUs were found to be more abundant in the MC-LR exposed group of BEBs compared with the no toxin control BEBs, Figure 6B. Specifically, 3 different *Sphigomonodales* were more abundant on the MC-LR exposed BEBs, Figure 6C. An uncultured *Sphigomonodales* bacterium KT182514.1.1452 was identified as more abundant in the MC-LR exposed group of BEBs compared with the no toxin control BEBs, Figure 6C. This OTU was identified as *Sphingorhabdus lacus* strain IMCC1753, using the BLASTn search engine. This is significant as *Sphingorhabdus* spp. are known microcystin degraders.^26,42,43^ The increased abundance of the 2 other *Sphigomonodales*, identified as *Sphingopyxis* sp., in the MC-LR exposed test samples is also indicative of the adaptation of the biochar microbiome for microcystin degradation. *Sphingopyxis* sp. has been shown to utilize the *mlr* operon to linearize and degrade MC-LR and may have been responsible for the transient linearized MC-LR breakdown product detected during the colonization phase, Figure 5B.^21,43,44^

### 3.4 Challenging BEBs with Microcystin Mixture

We have demonstrated that our BEBs are effective MC-LR degraders However, over 310 naturally occurring, chemically distinct microcystins have been reported.^45^ Therefore, for BEBs to be a viable solution for the clean water crisis, they need to be versatile in their ability to degrade microcystins.

**Figure 7:**
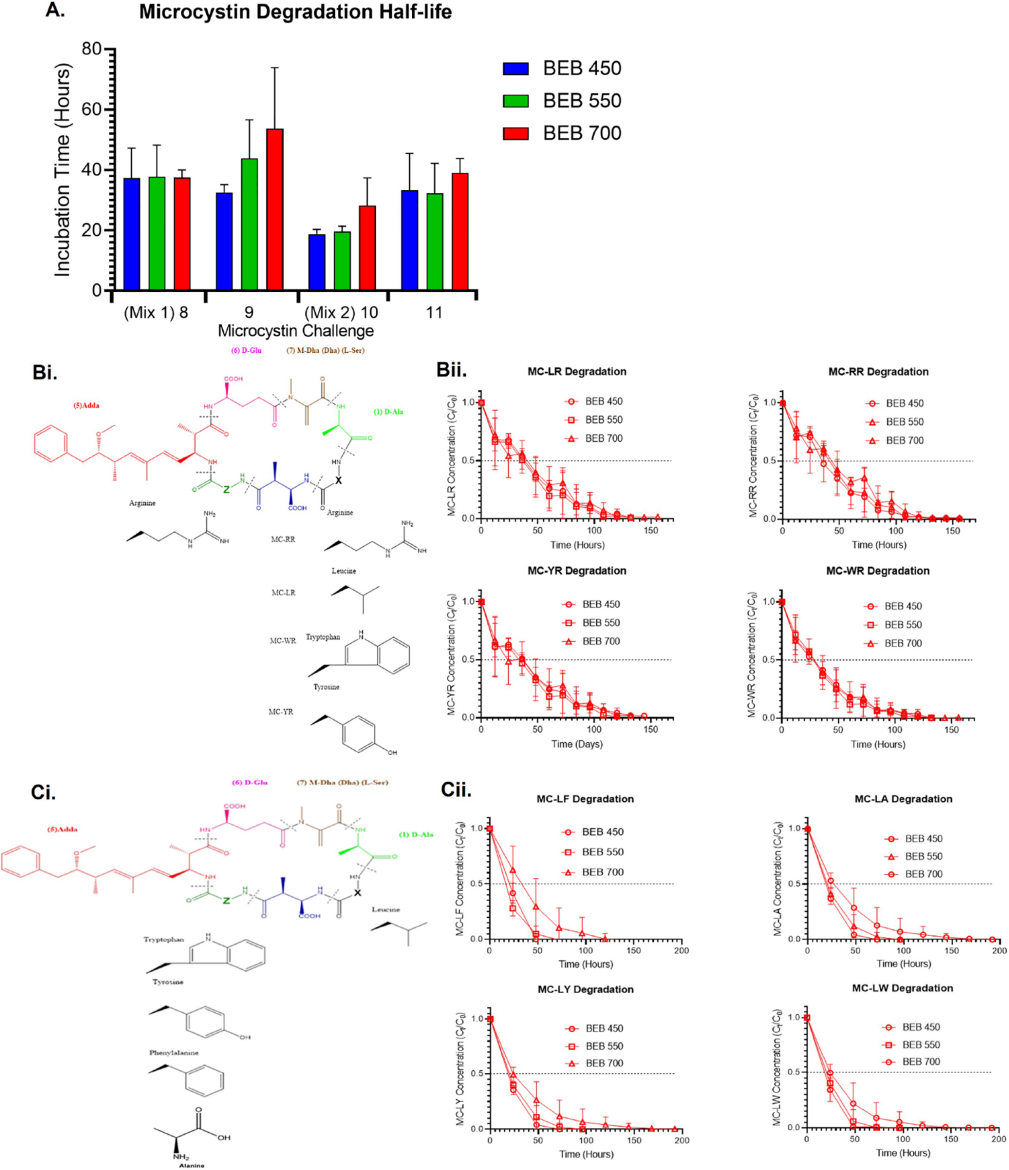
BEB Microcystin degradation. (A) Challenge 8–11, microcystin biodegradation half-life, in the presence of BEB. (Bi) Chemical structures of the challenge 8 microcystins; MC-LR, -RR, -YR, and -WR. (Bii) Challenge 8, microcystin concentrations monitored by UPLC-PDA-MS/MS to assess the rate of BEB biodegradation. (Ci) Chemical structures of the challenge 10 microcystins; MC-LF, -LA, -LY, and -LW. (Cii) Challenge 10, microcystin concentrations monitored by UPLC-PDA-MS/MS to assess the rate of biodegradation by the BEBs. Error bars represent the standard deviation *n* = 3.

BEBs from challenge 7 were aseptically transferred into fresh flasks containing sterilized lake water artificially contaminated with mixtures of chemically distinct microcystins (challenge 8–10). They were exposed to 2 different microcystin mixtures with a single MC-LR (5 μg/mL) checkpoint challenge between the 2 assays, to ensure that BEB functionality for single toxin degradation remained consistent. Initially, BEBs were challenged with a mixture of MC-LR (1.25 μg/mL), -RR (1.25 μg/mL), -YR (1.25 μg/mL), and -WR (1.25 μg/mL) (challenge 8), where the amino acid at position 2 is variable, Figure 7Bi. Despite the increased complexity, all BEBs were capable of degrading these microcystins, Figure 7A,Bii. The degradation rate was roughly three times slower than that observed for earlier MC-LR challenges. It is hypothesized that this is due to the increased chemical complexity of adding multiple microcystins. Proceeding with this, the BEBs were challenged with a mixture of MC-LF (1.25 μg/mL), -LA (1.25 μg/mL), -LY (1.25 μg/mL), and -LW (1.25 μg/mL) (challenge 10), where the amino acid at position 4 is varied, Figure 7Ci. The best-studied microcystin degradation pathway is encoded by the *mlr* operon. The first step of this pathway is the linearization of the microcystin by cleavage of the bond between amino acids 4 and 5.^46^ It was hypothesized that alteration of the amino acid at position 4 may inhibit the degradation process, therefore increasing the difficulty of the microcystin degradation challenging for the BEBs. Unexpectedly, the microcystin degradation half-life of challenge 10 was ca. 25–49% faster in comparison with challenge 8, Figure 7A,Bii. This may be explained by some of the *mlr-*independent microcystin degradation pathways that are known to exist but their mechanisms are less well understood.^21^

### 3.5 Challenging BEBs with Cyanobacteria

**Figure 8:**
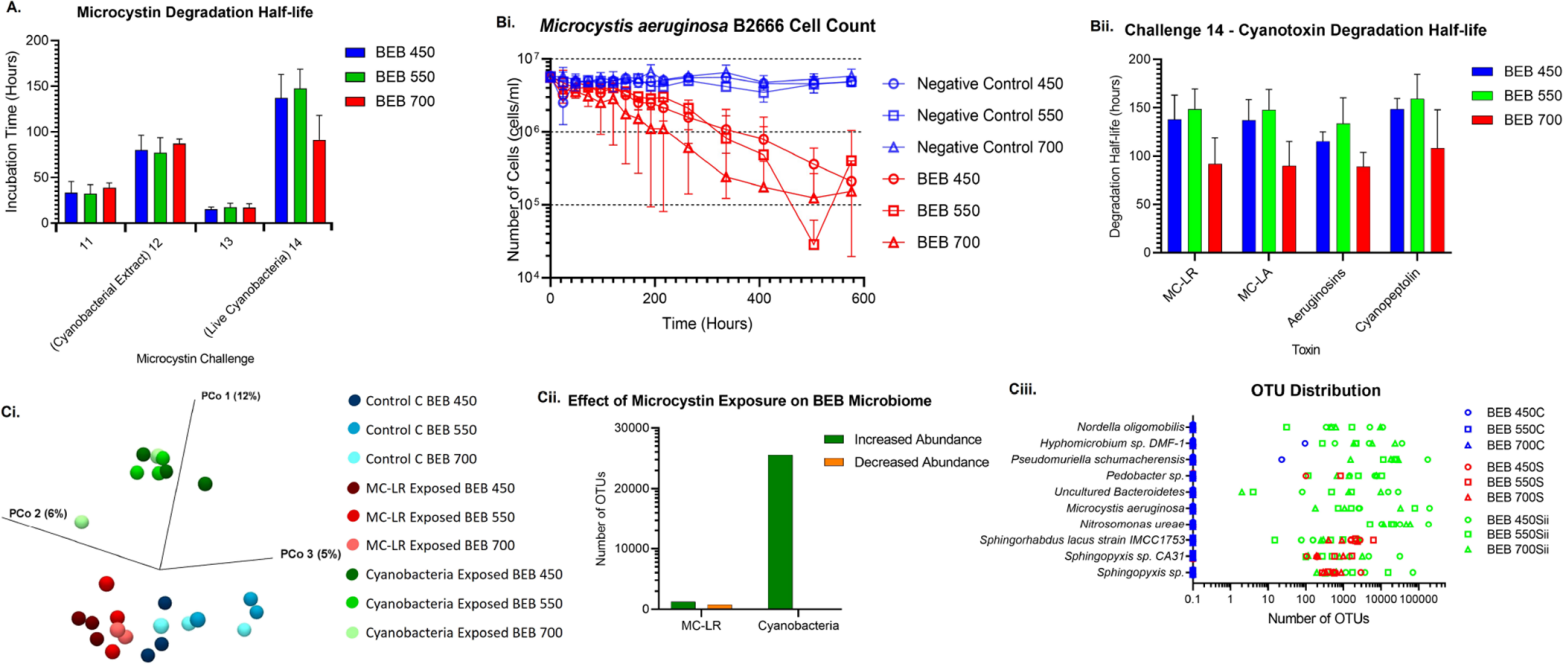
BEB cyanobacterial removal and microcystin degradation. (A) Challenge 11–14, MC-LR, and MC-LA biodegradation half-life in the presence of BEB. (Bi) Challenge 14, BEB removal of live cyanobacteria cells (*M. aeruginosa* B2666). (Bii) Challenge 14, BEB microcystin (MC-LR and -LA), putative aeruginosin, and cyanopeptolin 1020 degradation half-life. (Ci) Bray–Curtis principal coordinate analysis displays the species divergence between different BEB ecosystems. (Cii) Differential abundance analysis which displays the number of operational taxonomic units (OTUs) that are at-least 100-fold change in abundance on the surface of the BEB MC-LR (S) or cyanobacteria (Sii) exposed samples compared to the control samples (C). (Ciii) Distribution of some of the most abundant OTUs identified on the surface of the different biochar. Error bars represent the standard deviation *n* = 3.

All BEBs performed well when challenged with mixtures of chemically distinct microcystin compounds; therefore, the challenge was increased by exposing the BEBs to 25% *M. aeruginosa* B2666 cell lysate (dry weight 5.34 mg/mL), containing MC-LR (1.3 μg/mL) and -LA (0.3 μg/mL) (challenge 12), Supporting Information Table S4. Even in this complex environment, containing thousands of different molecules, complete microcystin removal was detected for all BEBs, with microcystin degradation half-lives of 77–87 h and 90% removal after 264 h incubation, this system still outperforms sand filtration and BAC, Figure 8A.^13,21^ As predicted in this biologically complex environment, the microcystin degradation rate is slower, ca. 7-fold slower than that observed for MC-LR alone (challenge 2–7), Figure 5A. It was also noted that a ca. 2-fold lower degradation rate of MC-LA than that of MC-LR was also observed, Supporting Information Figure S12. This may be due to variations in how these molecules are displayed within the molecularly more diverse and complex cellular lysate compared to the purified compounds used in previous challenges. The different molecules in cyanobacterial lysate may also compete with our target compounds for adsorption sites on the biochar surface, leading to increased mass transfer limitations.

The purpose of developing this technology is so that it can be used as a sustainable and economical solution for the sanitation of household drinking water. Even in this complex environment containing thousands of different molecules, complete microcystin removal releases intracellular toxins that are normally released upon cell death/lysis, often during ingestion by animals.^10,42,47,48^ Therefore, for the BEBs to effectively cleanse water supplies of microcystins, they must be able to remove live cyanobacterial contamination from the water source as well as microcystins.

To simulate a cyanobacterial bloom, that might be encountered in contaminated water, the BEBs were challenged with 5.5 × 10^6^ cells/mL live *M. aeruginosa* B2666 cells, producing the toxins MC-LR (0.4 μg/mL), MC-LA (0.16 μg/mL), aeruginosins, and cyanopeptolin (challenge 14). After 24 days of incubation, a 1.6–1.9 log reduction in the number of *M. aeruginosa* B2666 cells was observed, with a microcystin half-life of 92–148 h, Figure 8Bi. On closer analysis of individual toxin concentrations, it was found that not only were the BEBs degrading microcystin compounds (MC-LR and MC-LA), but also chemically and structurally distinct cyanotoxins (aeruginosins and cyanopeptolin), Figure 8Bii. The rate of degradation was similar for all cyanotoxins detected; however, BEB 700 was found to outperform the BEB 450 and BEB 550, Figure 8Bii. A more rapid reduction in the cell numbers of *M. aeruginosa* B2666 was also observed for BEB 700, with the highest log reduction of 1.9 after 24 days, Figure 8Bi. By the end of challenge 14, the same BEBs had been used to degrade microcystins for 11 months, indicating the long-lasting efficacy of this technology. It is also noted that although the assay was stopped after 11 months (14 challenges), there were no indications that the BEB efficacy was reducing, and it is hypothesized that BEBs could have very long functional life spans offering a considerable advantage over adsorption-based solutions.

On completion of challenge 14, 16S metagenomic analysis of the test samples was again conducted. This would allow us to determine whether further changes in the BEB microbiome could be detected after exposure to a broader range of compounds, cyanotoxins, and live cyanobacteria, Figure 8C. As expected, all BEBs were found to support diverse microbial communities. On comparison of these test samples (after challenge 14) with the previous naive and MC-LR exposed samples (taken after challenge 4), an increased divergence of the microbiome was detected, indicating that exposure to a broader range of compounds, cyanotoxins, and live cyanobacteria has altered the BEB microbial community, Figure 8Ci. Comparison of the abundance of individual OTUs confirmed this hypothesis, with ca. 25,000 OTUs found to be more abundant in the cyanobacteria-exposed group of BEBs compared with the no toxin control BEBs, Figure 8Bii. Unsurprisingly the cyanobacteria *M. aeruginosa* was detected after exposure to live cyanobacteria, Figure 8Ciii. The cyanobacteria used to artificially spike our freshwater during challenges 12 and 14 was *M. aeruginosa*; therefore, the abundance of this OTU after cyanobacterial exposure is attributed to our cyanobacterial inoculum, Supporting Information Table S4.

The abundance of *Sphingopyxis* sp. was again increased after cyanobacteria exposure (challenge 14), Figure 8Ciii. This is an important indication that the BEB microbiome is still primed for microcystin degradation even after 11 months of continual use and exposure to multiple cellular components and microcystins.

One of the new species identified as more abundant after cyanobacterial exposure was *Nitrosomonas ureae* (challenge 14), Figure 8Ciii. This species oxidizes ammonia to nitrite as a source of energy and can use urea as an alternative nitrogen source and is generally found in habitats where there is an abundance of protein decomposition.^49^ Therefore, the increased abundance of this organism may be explained by the increased abundance of cellular material during challenges 12 and 14. The release of nitrite into freshwater may increase the chances of further algal blooms. The increased abundance of *Hyphomicrobium* sp. DMF-1 (Figure 8Ciii), identified as a denitrifier, may counterbalance elevated nitrite as it has been shown to reduce nitrite concentrations in wastewater.^50,51^

*Nordella oligomobilis* was also found to be more abundant after cyanobacterial exposure, Figure 7Ciii. This organism was originally isolated using amoebal coculture.^52^ Little information could be found about this species although it is a member of the *Rhizobiales* order, synonymous with symbiotic nitrogen fixation with their plant hosts.^53^ Another of the OTUs identified as more abundant after cyanobacterial exposure was *Bacteroidetes* sp. These organisms play a role in the degradation of complex biopolymers and are often found in high abundance during periods of cyanobacterial bloom and in the presence of high quantities of DOC, therefore, could be playing a role in the degradation of the cyanobacterial cellular components.^54^

We have demonstrated that the natural freshwater microbiome can adapt to the degradation of chemically distinct cyanotoxins even in a nutrient-rich environment containing cyanobacterial cellular components. This technology has the potential to alleviate drinking water availability stresses in many affected areas of the world, especially in rural areas, where home-scale and community-scale biochar production techniques can be used with locally available resources, helping improve the quality of water for safe human consumption.^55^ We envisage that BEBs could be particularly useful for the treatment of drinking water with slow flow rates and high residence times, such as in communities where drinking wells are a water source. In addition, the use of local resources, such as coconut shells as biogenic waste to produce biochar, provides a readily available, low-cost, sustainable product. Moreover, coconut shell biochar has the necessary strength and durability to sustain long-term biodegradation in water. With around 70 billion coconuts produced globally per annum, utilizing the coconut shell reduces industrial waste and creates opportunities for a new economy.^56^ Local communities will benefit from better use of agricultural waste to produce biochar, which after its end-of-life in water treatment can be used as a soil amendment, as biochar also offers an estimated soil carbon sequestration potential of up to 6.6 Gt CO_2_ eq/year.^18,57^ BEBs have the potential to be applied to the remediation of other freshwater pollutants of emerging concern, such as pharmaceuticals and pesticides. This water treatment solution will have wide application, especially for low- and middle-income countries, and will contribute to achieving UN SDG6 while embracing the philosophy of the United Nations World Water Development Report, which emphasizes the benefits of 'Nature-Based Solutions for Water’.

## Data Availability

All data is available in the manuscript and Supporting Information.

## Supporting Information Available

Detailed information about the coconut shell pyrolysis conditions; biochar physical and chemical characterization; freshwater source chemical analysis; BEB challenge assay setup; cyanotoxin degradation profiles; statistical analysis of microcystin degradation rates; mass spectra analysis of *M. aeruginosa* B2666 extracts; and MC-LR degradation products (PDF)
