# Supplementary material for: Nature-Based Solution to Eliminate Cyanotoxins in Water Using Biologically Enhanced Biochar
Source: Environ Sci Technol. 2023 Oct 19;57(43):16372–85. doi: 10.1021/acs.est.3c05298 (PMC10620996; doi:10.1021/acs.est.3c05298)
Supplement: Supplementary file 1 — es3c05298_si_001.pdf [file es3c05298_si_001.pdf]

Supporting Information for

Nature-based solution to eliminate cyanotoxins in water using biologically enhanced biochar

Jane Moore[1*]†, Anjali Jayakumar[2,3]†, Sylvia Soldatou [1,4], Ondřej Mašek[3], Linda A Lawton[1], Christine Edwards[1]

Corresponding author: j.moore10@rgu.ac.uk

†-These authors contributed equally to this work

Summary: 26 pages, 7 tables, 18 figures

The PDF file includes:

Supporting information containing Fig. S1 to S18 and Tables S1 to S7, where SI Table S1 details the coconut shell pyrolysis conditions, SI Table. S5–S6 & Fig. S13 -S14 the biochar physical and chemical characterisation, SI Table. S2 the freshwater source chemical analysis, SI Fig. S1 & SI Table. S3-S4 BEB challenge assay conditions, SI Table. S7, Fig. S9, S10, S12 & S15-S18 cyanotoxin degradation profiles, SI Fig. S6-S8 statistical analysis of microcystin degradation rates, SI Fig. S2-S5 mass spectra analysis of *M. aeruginosa* B2666 extracts and SI Fig. S11 MC-LR degradation products.

Table. S1 - Coconut shell biochar pyrolysis conditions

Coconut shell biochar	HTT (℃)	Heating Rate (℃/min)	Residence Time	Carrier gas (N_2) flow rate (L/min)
COCO 450	450	25	45	0.35-0.4
COCO 550	550	25	30	0.35-0.4
COCO 700	700	25	30	0.35-0.4

HTT- Highest treatment Temperature, Residence Time- Time for which the coconut shells remain at HTT

Table S2 - Chemical Analysis of Rescobie Loch Water. Collected 13 January 2021. Water chemical analysis performed by the James Hutton Institute. Error represents the standard deviation n=3.

Chemical Test	Concentration (mg/L)
Total Organic Carbon	4.19 ± 0.14
Dissolved Organic carbon	4.51 ± 0.40
Total Nitrogen	4.37 ± 0.39
NH_4-N	0.68 ± 0.38
Total organic Nitrogen	3.69 ± 0.05
Organic Nitrogen	0.00 ± 0.05
Phosphorous	0.03 ± 0.00
PO_4 -P	0.00 ± 0.00
Organic Phosphorous	0.02 ± 0.00
Chemical Oxygen Demand	18.33 ± 1.93
Biological Oxygen Demand	<4

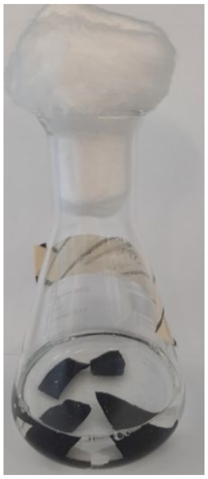

Fig. S1 – Biologically enhanced biochar nature-based solution for microcystin removal from contaminated water.
Lab scale proof-of-concept study displaying BEBs in lake water spiled with 5 µg/ml MC-LR.

Table S3 – Summary of Sample Flask Set-up for each Challenge Assay 1-7

Challenge	Flask Contents	Sample Set			
		Control A	Control B	Control C	Test Samples
Colonisation	Rescobie Loch Water	Sterile	Non-sterile, set up within 48 hours of collection	Non-sterile, set up within 48 hours of collection	Non-sterile, set up within 48 hours of collection
	5-7 Coconut Biochar Pellets	P	X	P	P
	Microcystins	5 µg/ml MC-LR	5 µg/ml MC-LR	X	5 µg/ml MC-LR
1	Rescobie Loch Water	Sterile	Discontinued	Sterile	Sterile
	5-7 Coconut Biochar Pellets	From challenge 0	Discontinued	From challenge 0	From challenge 0
	Microcystins	5 µg/ml MC-LR	Discontinued	X	5 µg/ml MC-LR
2	Rescobie Loch Water	Sterile		Sterile	Sterile
	5-7 Coconut Biochar Pellets	From challenge 1		From challenge 1	From challenge 1
	Microcystins	5 µg/ml MC-LR		X	5 µg/ml MC-LR
3	Rescobie Loch Water	Sterile		Sterile	Sterile
	5-7 Coconut Biochar Pellets	From challenge 2		From challenge 2	From challenge 2
	Microcystins	5 µg/ml MC-LR		X	5 µg/ml MC-LR
4	Rescobie Loch Water	Sterile		Sterile	Sterile
	5-7 Coconut Biochar Pellets	From challenge 3		From challenge 3	From challenge 3
	Microcystins	5 µg/ml MC-LR		X	5 µg/ml MC-LR
5	Rescobie Loch Water	Sterile		Discontinued	Sterile
	5-7 Coconut Biochar Pellets	From challenge 4		Discontinued	From challenge 4
	Microcystins	5 µg/ml MC-LR		Discontinued	5 µg/ml MC-LR
6	Rescobie Loch Water	Sterile			Sterile
	5-7 Coconut Biochar Pellets	From challenge 5			From challenge 5
	Microcystins	5 µg/ml MC-LR			5 µg/ml MC-LR
7	Rescobie Loch Water	Sterile			Sterile
	5-7 Coconut Biochar Pellets	From challenge 6			From challenge 6
	Microcystins	5 µg/ml MC-LR			5 µg/ml MC-LR

* All purified microcystins purified as per Enzo Life Sciences

Table S4 – Summary of Sample Flask Set-up for each Challenge Assay 8-14

Challenge	Flask Contents	Sample Set			
		Control A	Control B	Control C	Test Samples
8	Rescobie Loch Water	Sterile			Sterile
	5-7 Coconut Biochar Pellets	From challenge 7			From challenge 7
	Microcystins	MC-LR, -RR, -YR & -WR, 1.25 µg/ml each			MC-LR, -RR, -YR & -WR, 1.25 µg/ml each
9	Rescobie Loch Water	Sterile			Sterile
	5-7 Coconut Biochar Pellets	From challenge 8			From challenge 8
	Microcystins	5 µg/ml MC-LR			5 µg/ml MC-LR
10	Rescobie Loch Water	Sterile			Sterile
	5-7 Coconut Biochar Pellets	From challenge 9			From challenge 9
	Microcystins	MC-LA, -LF, -LY & -LW 1.25 µg/ml each			MC-LA, -LF, -LY & -LW 1.25 µg/ml each
11	Rescobie Loch Water	Sterile			Sterile
	5-7 Coconut Biochar Pellets	From challenge 10			From challenge 10
	Microcystins	5 µg/ml MC-LR			5 µg/ml MC-LR
12	Rescobie Loch Water	Sterile			Sterile
	5-7 Coconut Biochar Pellets	From challenge 11			From challenge 11
	Microcystins	1.34 mg/ml *Microcystis aeruginosa* B2666 extract			1.34 mg/ml *Microcystis aeruginosa* B2666 extract
13	Rescobie Loch Water	Sterile			Sterile
	5-7 Coconut Biochar Pellets	From challenge 12			From challenge 12
	Microcystins	5 µg/ml MC-LR			5 µg/ml MC-LR
14	Rescobie Loch Water	Sterile			Sterile
	5-7 Coconut Biochar Pellets	From challenge 13			From challenge 13
	Microcystins	5.8×10^6 cells/ml *Microcystis aeruginosa* B2666			5.8×10^6 cells/ml *Microcystis aeruginosa* B2666

* All purified microcystins purified as per Enzo Life Sciences

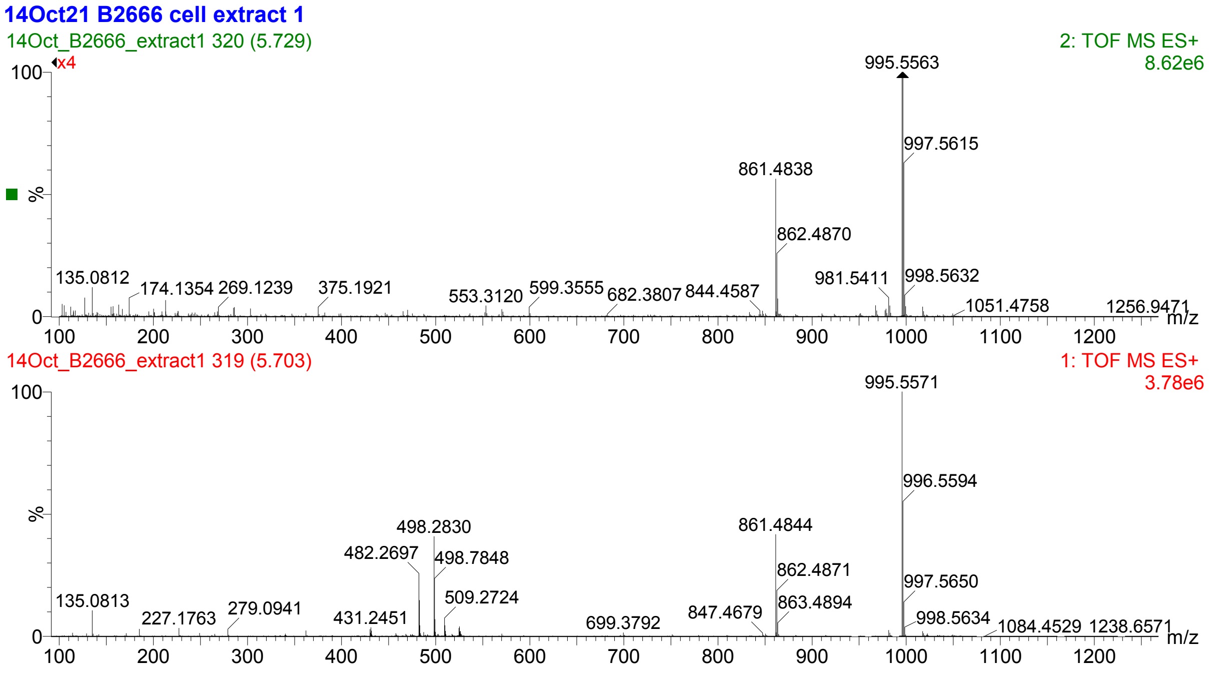

Fig. S2 – Mass spectra of MC-LR extracts of *M. aeruginosa* B2666.
The top spectra displays the high energy and the bottom spectra the low energy mass spectra for MC-LR in extracts of *M. aeruginosa* B2666.

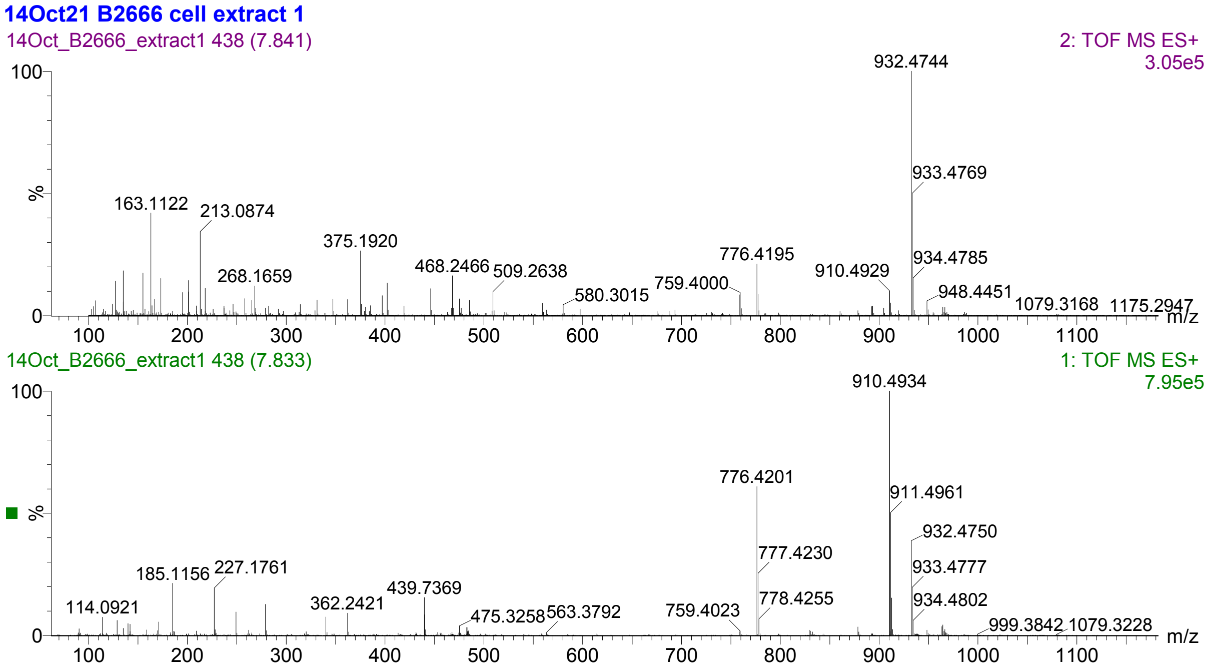

Fig. S3 – Mass spectra of MC-LA extracts of *M. aeruginosa* B2666.

The top spectra displays the high energy and the bottom spectra the low energy mass spectra for MC-LA in extracts of *M. aeruginosa* B2666.

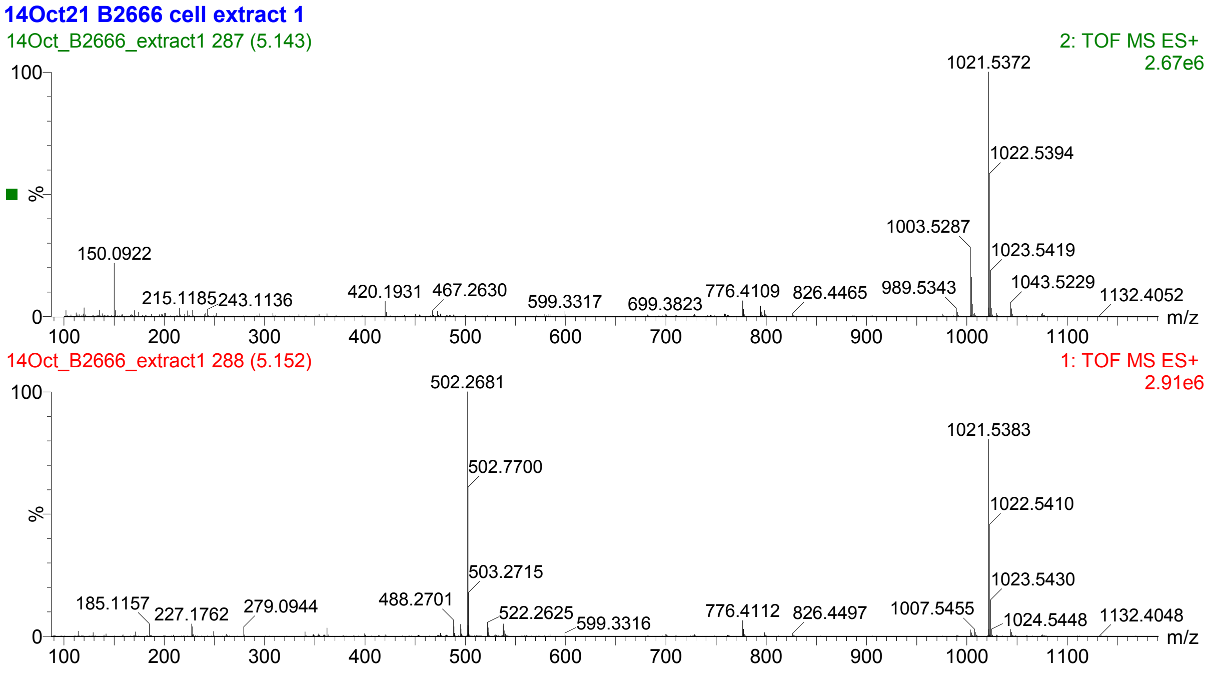

Fig. S4 – Mass spectra of cyanopeptolin 1012 extracts of *M. aeruginosa* B2666.
The top spectra displays the high energy and the bottom spectra the low energy mass spectra of cyanopeptolin 1020 in extracts of *M. aeruginosa* B2666.

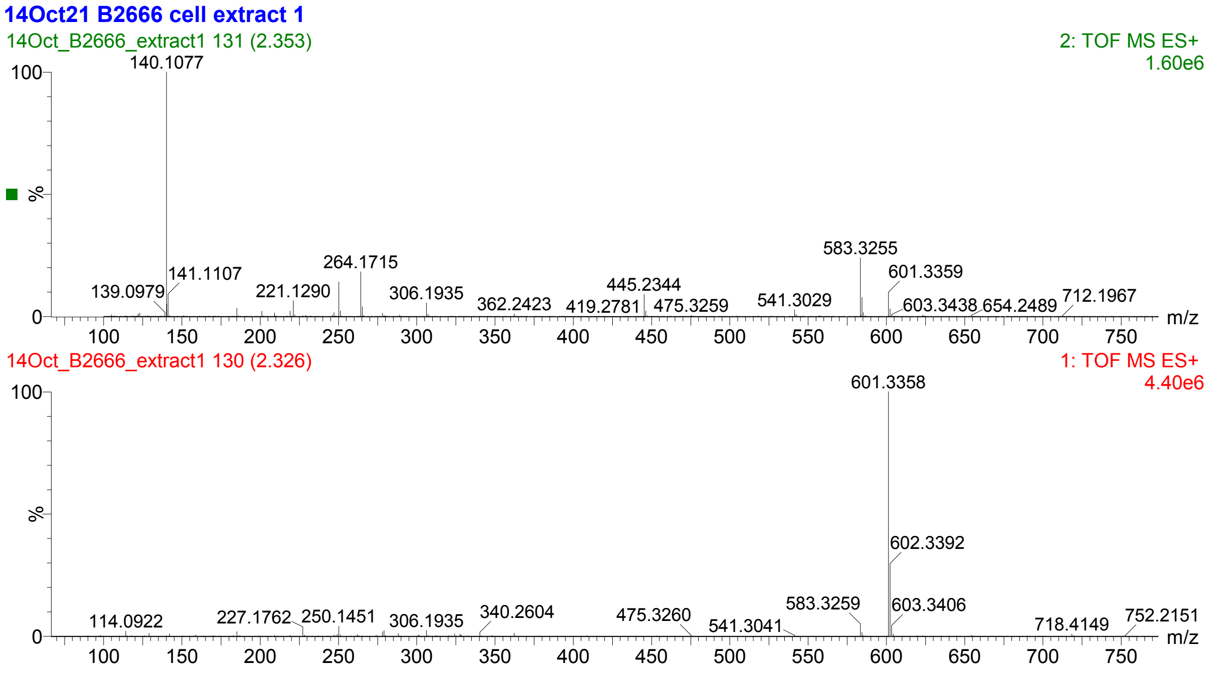

Fig. S5 – Mass spectra of putative aeruginosin extracts of *M. aeruginosa* B2666.
The top spectra displays the high energy and the bottom spectra the low energy mass spectra putative aeruginosin in extracts of *M. aeruginosa* B2666.

Table. S5 - Properties of coconut shell biochar obtained from a batch scale pyrolysis unit. EC- Electrical conductivity, PAHs- Polycyclic Aromatic Hydrocarbons, SSA- Specific Surface Area, FC- Fixed Carbon, VM- Volatile Matter. Errors represent the standard deviation, [1]-n=4, [2]-n=3, [3]- n=2, FC- Fixed Carbon, VM- Volatile Matter, , % wt. d.b: Yields and composition of coconut shell biochar were calculated as a proportion of the mass of dry feed.

**Analysis**	**Component**	**Coconut Shell Biochar**		
		**COCO 450**	**COCO 550**	**COCO 700**
Pyrolysis Yield[1]	Biochar (wt. % d.b)	33.14 ±1.32	29.69 ± 0.28	27.58 ±0.8
Proximate Analysis[2]	FC (wt. % d.b)	78.19±0.51	82.36±0.83	88.35±0.36
	VM (wt. % d.b)	20.73±0.68	15.71±0.76	8.83±0.22
	Ash (wt. % d.b)	1.09±0.45	1.92±0.07	2.82±0.27
Elemental Analysis[1]	C (wt. % d.b)	79.84 ± 0.18	87.53 ± 0.30	90.22 ± 0.96
	H (wt. % d.b)	3.15±0.04	2.55±0.03	1.49±0.01
	N (wt. % d.b)	2.80±0.01	3.99±0.01	2.39±0.01
	O (wt. % d.b)	15.50±0.38	6.38±0.13	4.96±0.14
	O:C	0.15	.05	0.04
	H:C	0.47	0.35	0.20
Other physico-chemical	pH[3]	7.08 ± 0.06	7.495 ± 0.05	8.26 ± 0.06
	EC[3] (dSm^{-1})	249.5 ± 40.31	210.5 ± 29.49	385 ± 19.80
	Id/Ig	0.709	0.712	0.765
	US 16 EPA PAHs (mg/Kg)	3.88	5.83	0.338
	BET Specific Surface Area[3] (m^2/g)	18.03±5.80	194.33±7.28	338.54±21.36

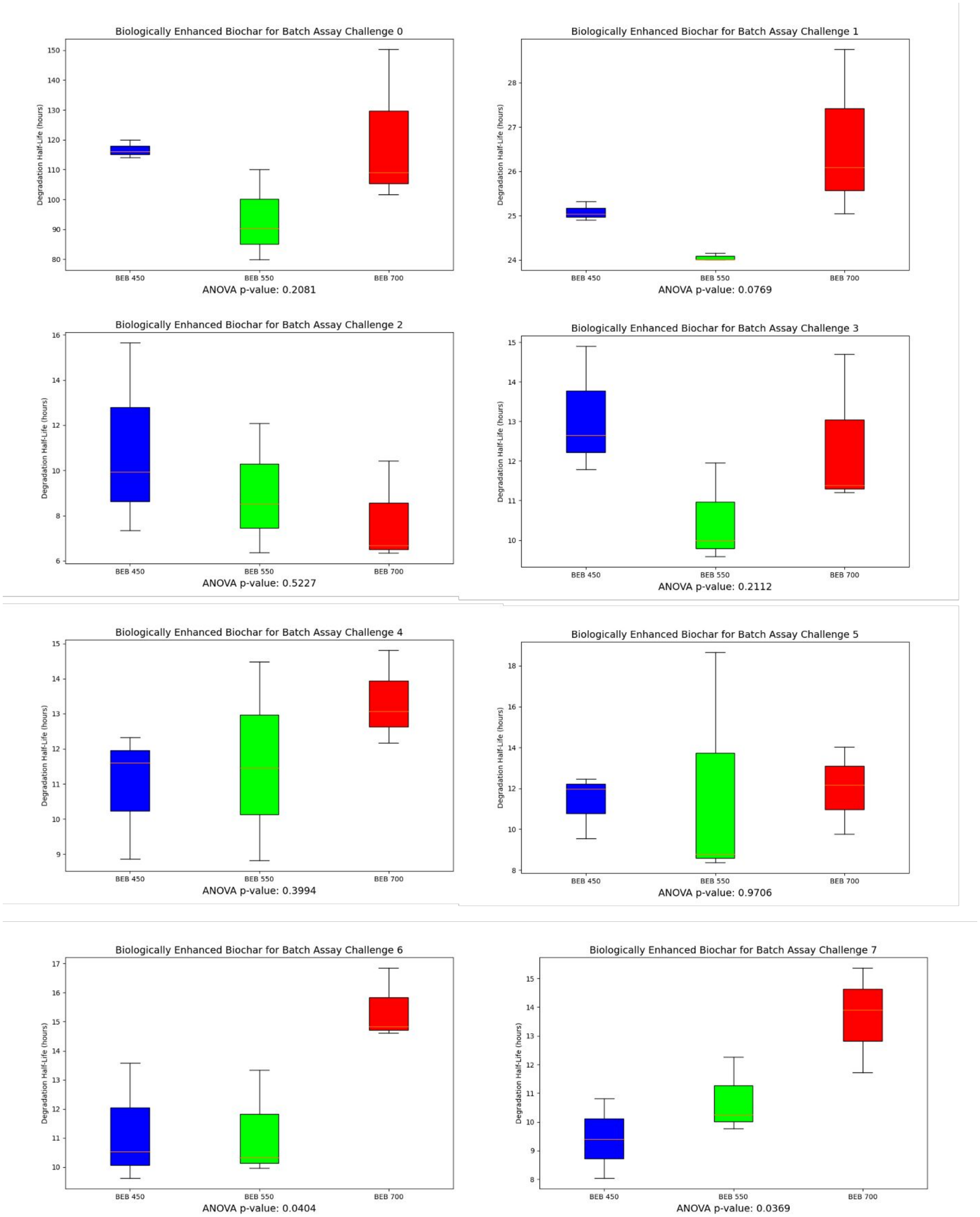

Fig. S6 - Box plots of degradation half-lives of BEB 450, BEB 550 and BEB 700 produced from batch-scale pyrolysis unit for challenge 0-7 with p-values obtained from One-way ANOVA tests.

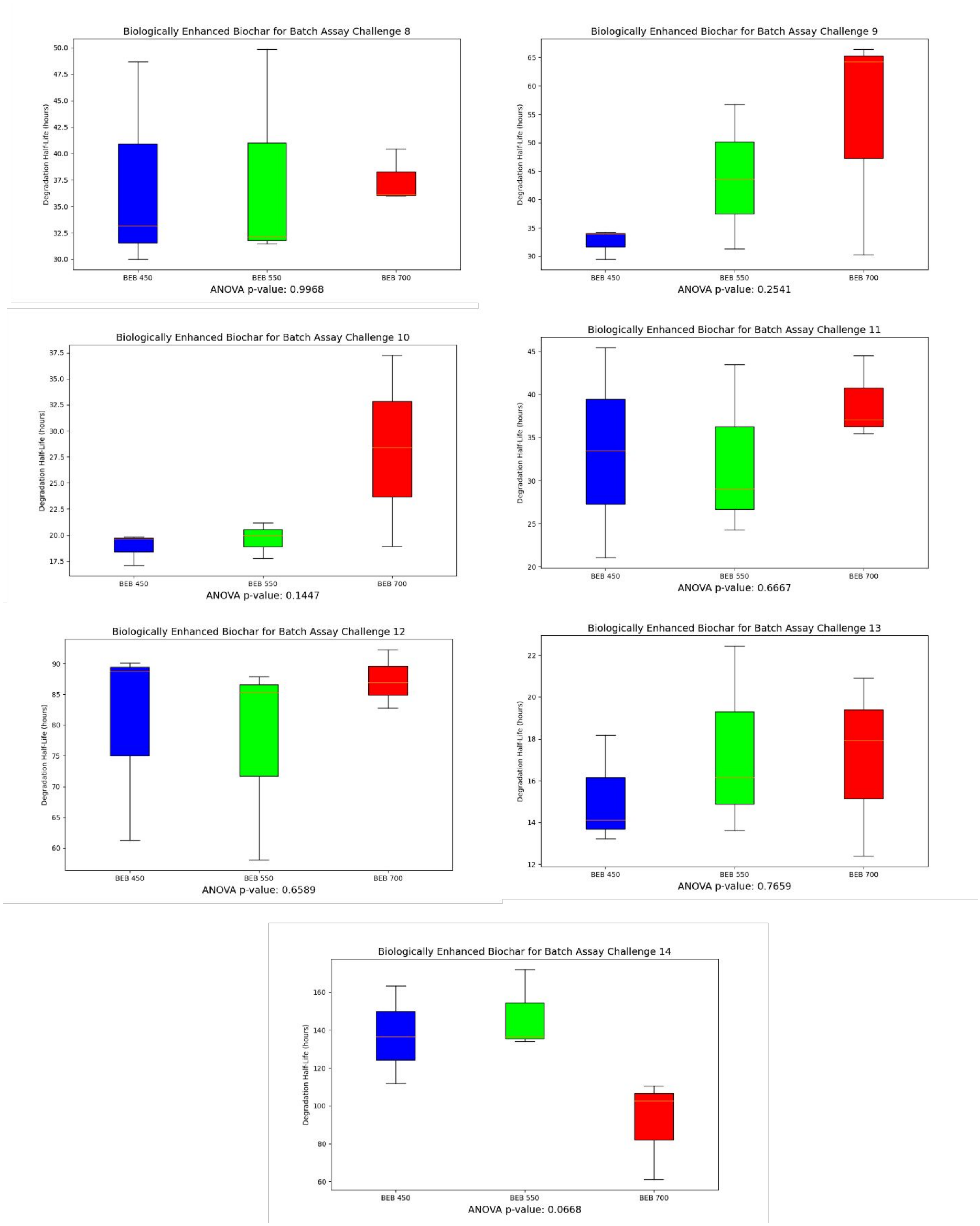

Fig. S7 - Box plots of degradation half-lives of BEB 450, BEB 550 and BEB 700 produced from batch-scale pyrolysis unit for challenge 7-14 with p-values obtained from One-way ANOVA tests.

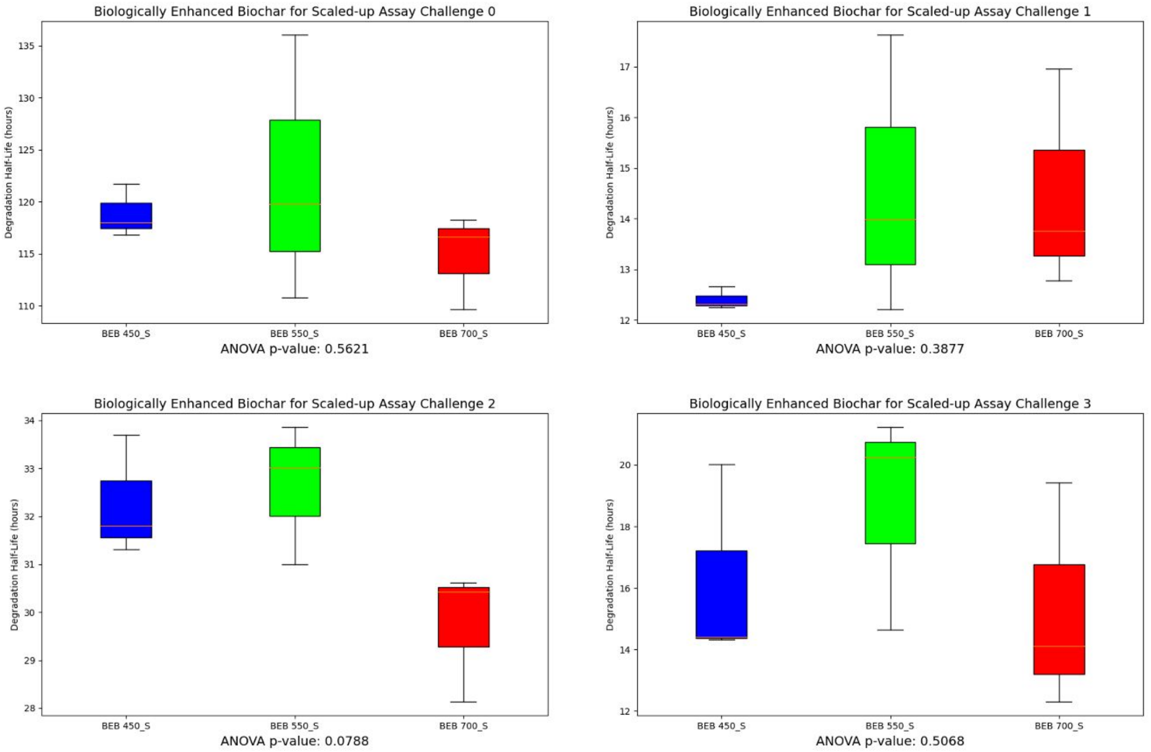

Fig. S8 - Box plots of degradation half-lives of biologically enhanced biochar produced from continuous-scale pyrolysis unit, represented as BEB 450_S, BEB 550_S and BEB 700_S, for challenge 0-3 with p-values obtained from One-way ANOVA tests.

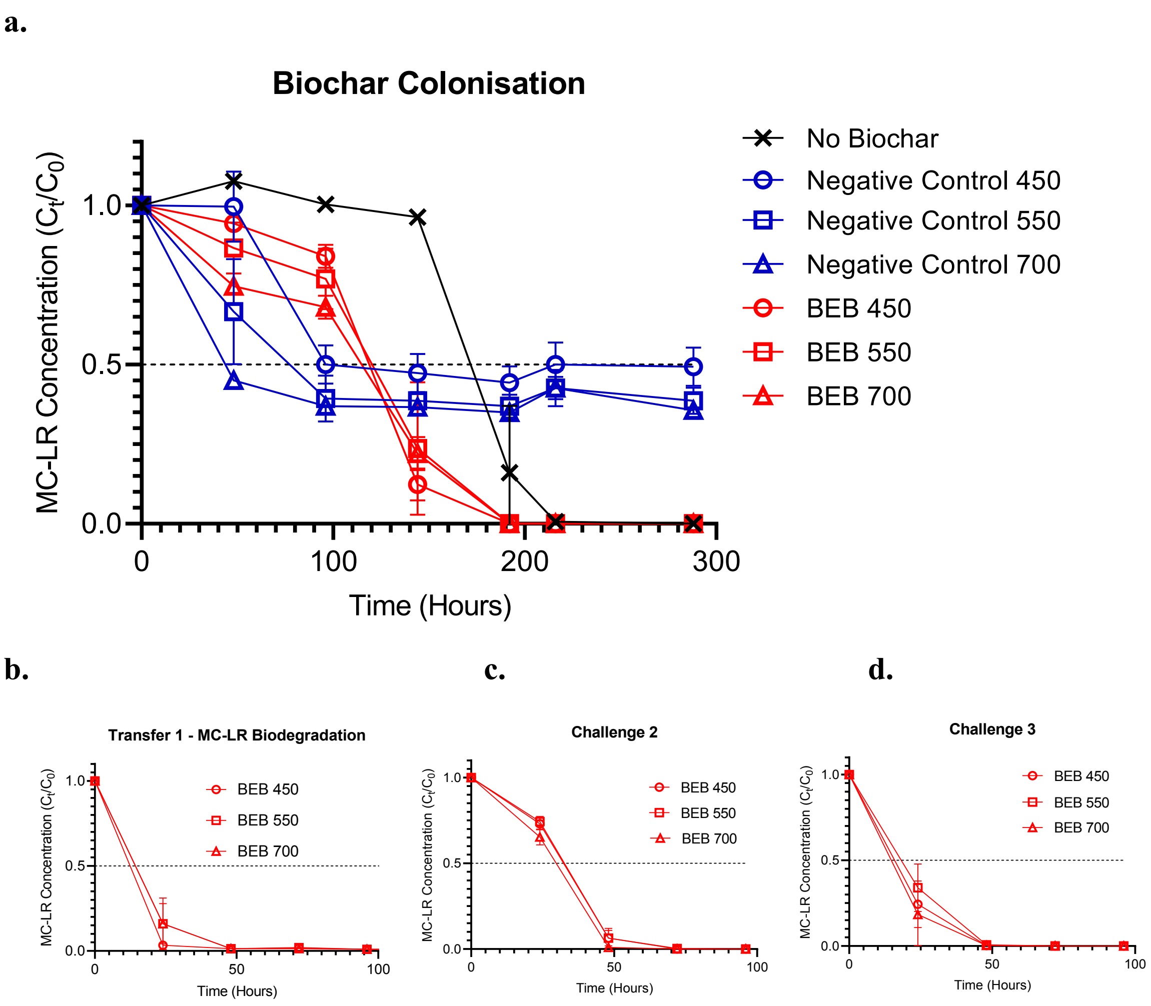

Fig. S9 – MC-LR degradation using biologically enhanced biochar, produced using a continuous scale pyrolysis unit

a. MC-LR removal from the contaminated lake water during the initial biochar colonisation stage when naturally occurring Rescobie Loch water microorganisms spontaneously start to colonise the surface of the biochar to form biologically enhanced biochar**.**

Negative controls, (blue lines) consist of coconut shell biochar and STERILE Rescobie Loch water, therefore, there are no microcystin biodegrading organisms present in this sample. No biochar, (black line) consists of non-sterile Rescobie Loch water, therefore, contains microcystin degrading organisms, but NO coconut shell biochar. BEBs, (red lines) contains coconut biochar and non-sterile Rescobie Loch water, therefore, microcystin degrading organisms will be present in these samples. **b.** Challenge 1: MC-LR, **c.** Challenge 2: MC-LR, **d.** Challenge 3: MC-LR. Where 450, 550 & 700 refers to the HTT pyrolysis temperature (℃) on synthesis of the coconut biochar. The microcystin concentration was monitored by UPLC-PDA-MS/MS to assess the rate of biodegradation by the biologically enhanced coconut biochar. Error bars represent the standard deviation, n=3.

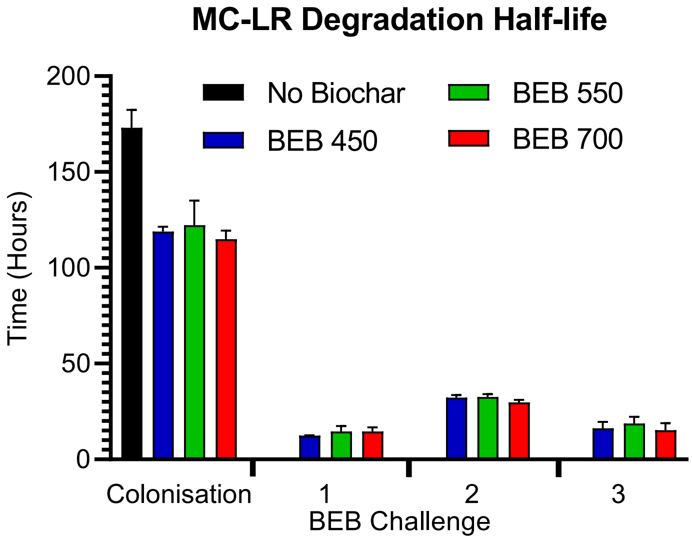

Challenge	Microcystin	Incubation Time Required for 50 % Microcystin Degradation (hours)		
		BEB 450	BEB 550	BEB 700
Colonisation	MC-LR	118.86 ± 2.56	122.18 ± 12.82	114.83 ± 4.56
1	MC-LR	12.41 ± 0.22	14.61 ± 2.76	14.50 ± 2.18
2	MC-LR	32.27 ± 1.26	32.63 ± 1.47	29.73 ± 1.38
3	MC-LR	16.25 ± 3.26	18.71 ± 3.56	15.28 ± 3.70

Fig. S10 - Microcystin biodegradation half-life, in the presence of biologically enhanced biochar, using a scaled-up coconut biochar production methodology.
The microcystin concentrations were monitored by UPLC-PDA-MS/MS to assess the rate of BEB biodegradation, using coconut shell biochar synthesized using a continuous scaled-up production methodology. The time taken for 50 % of the microcystins to be degraded was then calculated. 450, 550 & 700 refers to the HTT pyrolysis temperature (°C) on synthesis of the coconut biochar. Error bars represent the standard deviation, n=3.

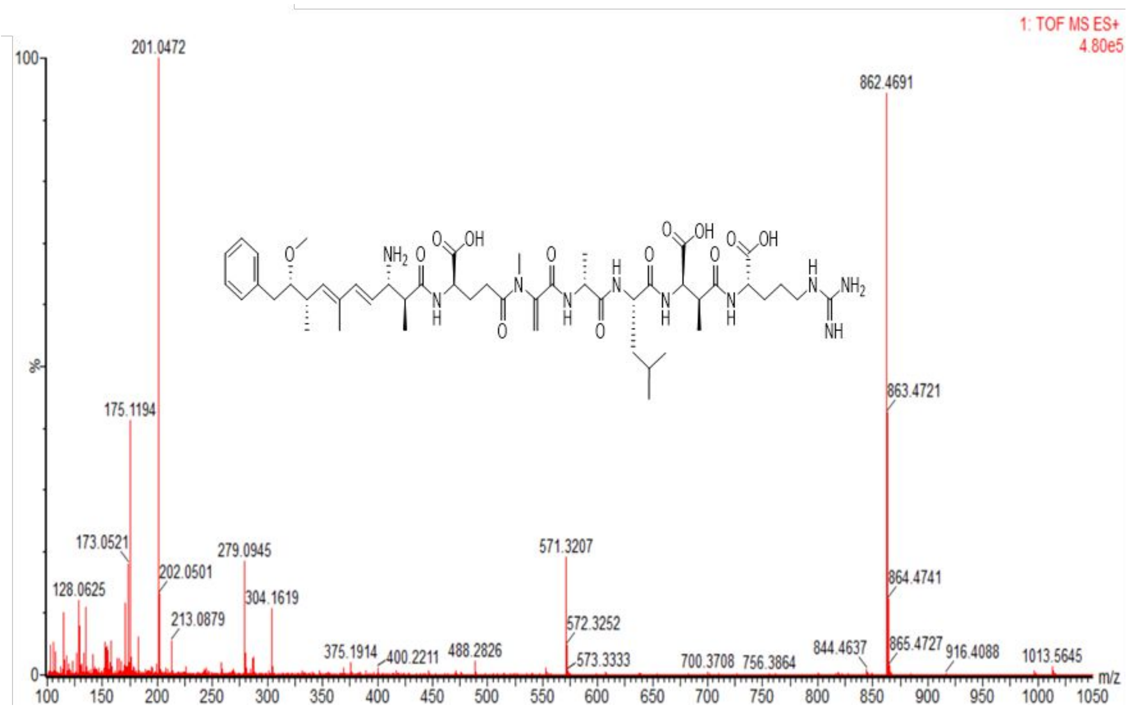

Fig S11 – MS spectrum of MC-LR degradation products.

Detected degradation intermediate of MC-LR was the linear peptide, acyclo MC-LR (NH2-Adda-Glu-Mdha-Ala-Leu-MeAsp-Arg-OH). Since the predominant ion was *m/z* 862.5 $[M - NH_2 - PhCH2CHOMe + H]^+$, extracted ion chromatograms at m/z 863 were used to monitor the degradation of this biodegradation intermediated.

Challenge 12 - Microcystin Degradation Half-life

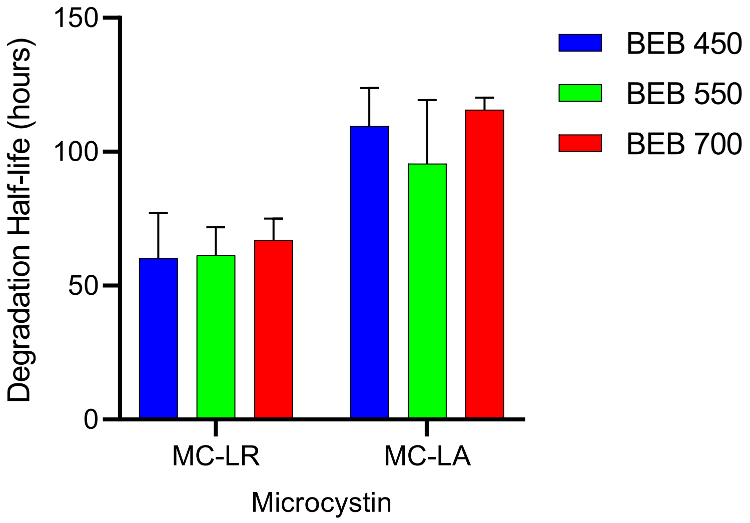

Microcystin	Incubation Time Required for 50 % Microcystin Degradation (hours)		
	**BEB 450**	**BEB 550**	**BEB 700**
MC-LR	60.17 ± 16.92	61.31 ± 10.47	66.94 ± 8.17
MC-LA	109.55 ± 14.21	95.56 ± 23.82	115.65 ± 4.55

Fig. S12 - Microcystin biodegradation half-life, in the presence of biologically enhanced biochar challenge 12: Cyanobacterial Extract.
The microcystin concentration was monitored by UPLC-PDA-MS/MS to assess the rate of biodegradation by the biologically enhanced coconut biochar. The time taken for 50 % of the each microcystin to be degraded was then calculated. BEB 450, 550 & 700 refers to the HTT pyrolysis temperature on synthesis of the coconut shell biochar. Error bars represent the standard deviation n=3.

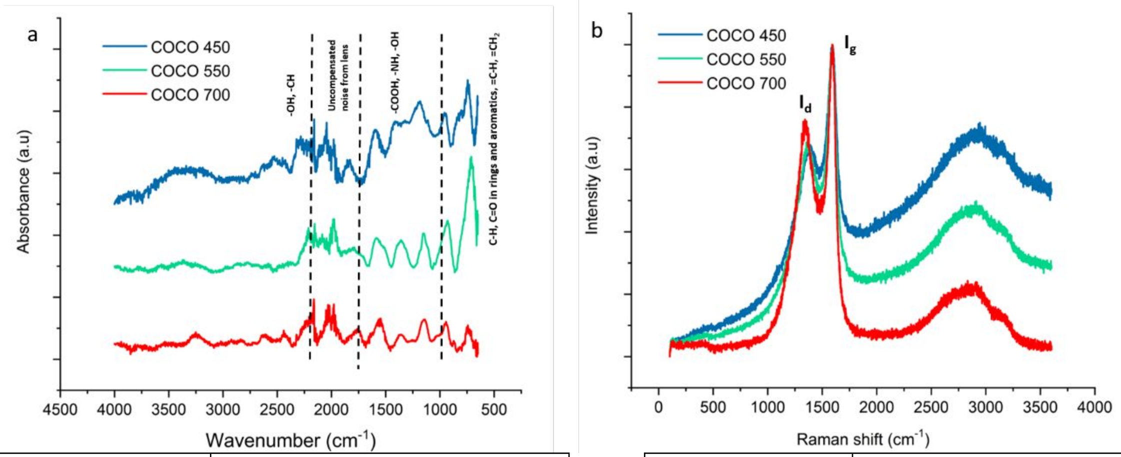

FTIR peak positions (cm^{-1})	Assignment
3200-3550	O-H (H-bonded)
2400-2800	C-H (stretching, carboxylic acid, aldehyde, CH_2, CH_3)
1650-1750	C=O (carbonyl groups), C=C
1500-1650	C=C, NH_2 scissoring, N-H bands in amines
1370-1420	OH-and C-O-H bending; CH_2 and CH_3 bending and deformation
1000-1200	O-C in esters, ether, alcohol
940-970	=C-H, =CH_2, C-O
700-800	C=C, CH in aromatics and rings
1900-2200	Uncompensated noise from lens

Raman peak positions (cm^{-1})	Assignment
1350-1370	D band, vibrations of in-plane sp2-bonded carbon with defects in their structure
1580-1600	G band, vibrations of in-plane graphitic sp2 carbon

Fig. S13 - FTIR and Raman spectra of coconut shell biochar obtained from batch scale pyrolysis unit
a. FTIR spectra of COCO 450, COCO 550 and COCO 700 and peak positions and surface functionality assignments**. b.** Raman spectra of COCO 450, COCO 550 and COCO 700 showing different Ig and Id peak intensities, positions and surface functionality assignments.

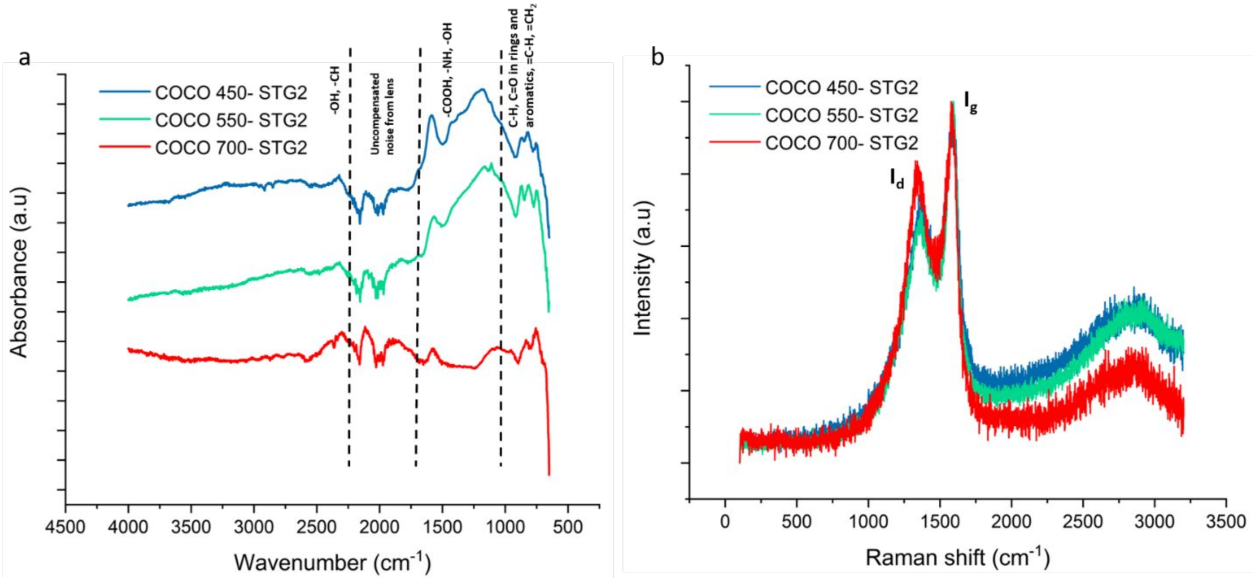

Fig. S14 - FTIR and Raman spectra of coconut shell biochar obtained from a continuous-scale (Stage 2) pyrolysis unit.

a. FTIR spectra of COCO 450-STG 2, COCO 550-STG2 and COCO 700-STG2 showing surface functionalities, **b.** Raman spectra of COCO 450-STG2, COCO 550-STG2 and COCO 700-STG2 showing different Ig and Id peak intensities.

Table. S6 - Properties of coconut shell biochar obtained from a continuous-scale (Stage 2) pyrolysis unit.
FC- Fixed Carbon, VM- Volatile Matter, Errors represent the standard deviation n= 4 , % wt. d.b: Yields and composition of coconut shell biochar were calculated as a proportion of the mass of dry feed FC- Fixed carbon, VM- Volatile matter

**Analysis**	**Component**	**Coconut Shell Biochar**		
		**COCO 450**	**COCO 550**	**COCO 700**
Pyrolysis Yield	Biochar (wt. % d.b)	30.83	29.54	25.06
Proximate Analysis	FC (wt. % d.b)	76.14 ± 1.33	85.10 ± 0.80	89.86 ± 0.4
	VM (wt. % d.b)	20.46 ± 1.42	11.07 ± 0.51	6.66 ± 0.66
	Ash (wt. % d.b)	3.40 ± 0.2	3.83 ± 0.72	3.49 ± 0.52
Elemental Analysis	C (wt. % d.b)	84.74 ± 5.33	87.17 ±0.29	86.17 ± 4.96
	H (wt. % d.b)	3.35 ± 0.19	2.74 ± 0.003	1.58 ± 0.14
	N (wt. % d.b)	0.40 ± 0.1	0.37 ± 0.03	0.32 ± 0.01
	O (wt. % d.b)	12.63 ± 0.20	6.16 ± 0.14	4.73 ± 0.04
	O:C	0.112	0.053	0.041
	H:C	0.474	0.377	0.22
Other physico-chemical properties	Id/Ig	0.708	0.729	0.838

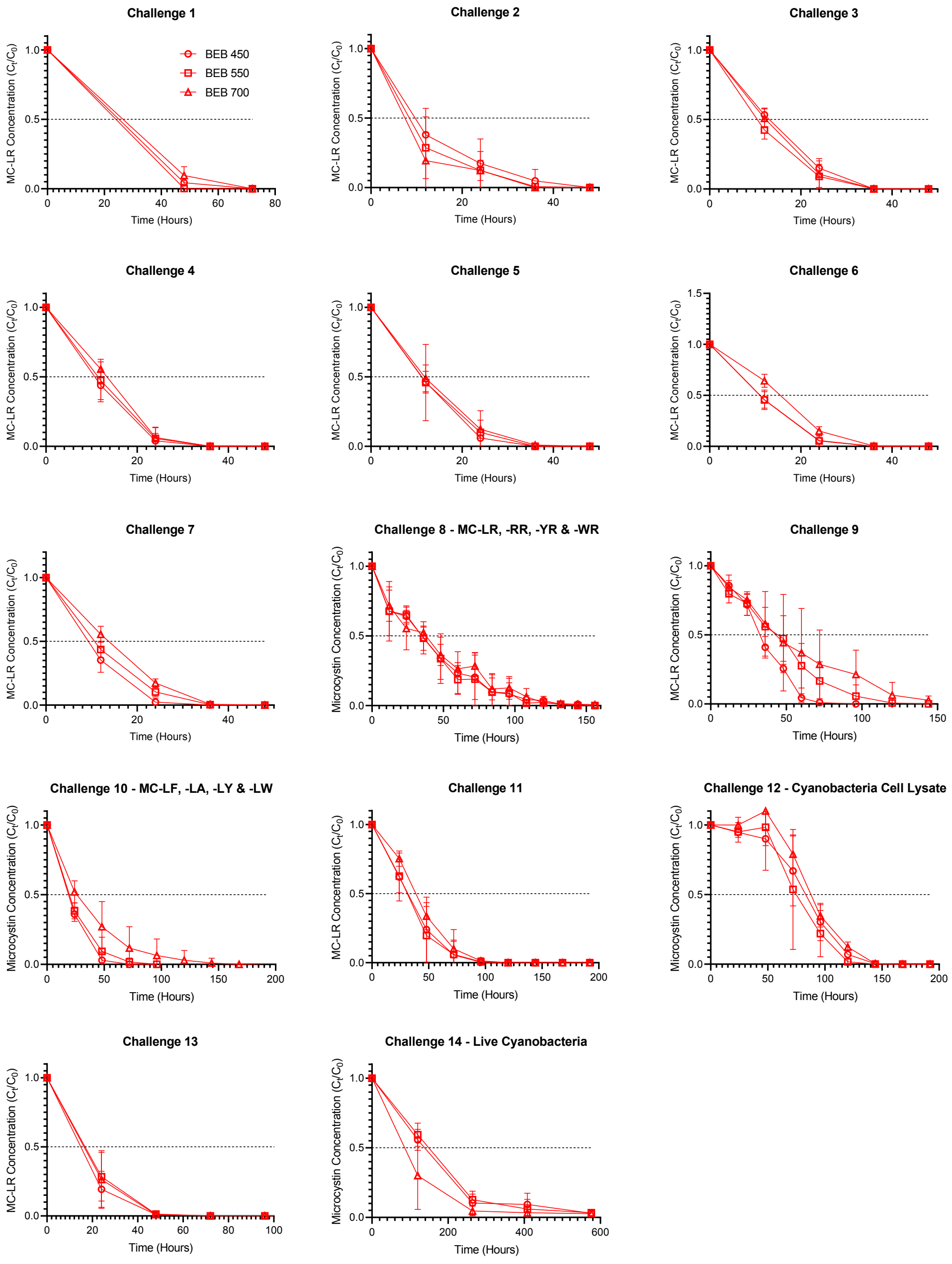

Fig. S15 - Microcystin degradation using biologically enhanced biochar.
The microcystin concentration for the biologically enhanced biochar (BEB) test samples was monitored by UPLC-PDA-MS/MS to assess the rate of biodegradation by the biologically enhanced coconut biochar. Challenge 1: MC-LR, Challenge 2: MC-LR, Challenge 3: MC-LR, Challenge 4: MC-LR, Challenge 5: MC-LR, Challenge 6: MC-LR, Challenge 7: MC-LR, Challenge 8: MC-LR, -RR, -YR & -WR, Challenge 9: MC-LR, Challenge 10: MC-LA, -LF, -LY & -LW, Challenge 11: MC-LR, Challenge 12: Cyanobacterial Extract; MC-LR & -LA, Challenge 13: MC-LR & Challenge 14: Live Cyanobacterial Cells; MC-LR & -LA. Where 450, 550 & 700 refers to the HTT pyrolysis temperature (℃) on synthesis of the coconut biochar. The microcystin concentration was monitored by UPLC-PDA-MS/MS to assess the rate of biodegradation by the biologically enhanced coconut biochar. Error bars represent the standard deviation, n=3.

Table S7- Microcystin biodegradation half-life, in the presence of biologically enhanced biochar.

The microcystin concentration was monitored by UPLC-PDA-MS/MS to assess the rate of biodegradation by the biologically enhanced coconut biochar. The time taken for 50 % of the microcystins to be degraded was then calculated. BEB 450, 550 & 700 refers to the HTT pyrolysis temperature on synthesis of the coconut shell biochar. Error represents the standard deviation n=3.

Challenge	Microcystins Present	Incubation Time Required for 50 % Microcystin Degradation (hours)		
		BEB 450	BEB 550	BEB 700
Colonisation	MC-LR	116.67 ±2.97	93.43 ± 15.33	120.35 ± 26.18
1	MC-LR	25.08 ± 0.21	24.06 ± 0.09	26.63 ± 1.91
2	MC-LR	10.97 ± 4.26	8.99 ± 2.88	7.82 ± 2.27
3	MC-LR	13.11 ± 1.61	10.51 ± 1.26	12.43 ± 1.97
4	MC-LR	10.93 ± 1.83	11.59 ± 2.84	13.35 ± 1.34
5	MC-LR	11.33 ± 1.56	11.94 ± 5.82	11.99 ± 2.14
6	MC-LR	11.24 ± 2.08	11.21 ± 1.85	15.43 ± 1.24
7	MC-LR	9.42 ± 1.39	10.76 ± 1.33	13.67 ± 1.84
8	MC-LR, -RR, -YR & -WR	37.27 ± 9.99	37.82 ± 10.43	37.52 ± 2.54
9	MC-LR	32.53 ± 2.66	43.91 ±12.73	53.65 ± 20.26
10	MC-LA, -LF, -LY & -LW	18.86 ± 1.53	19.63 ± 1.72	28.21 ± 9.16
11	MC-LR	33.34 ± 12.18	32.29 ± 9.96	39.01 ± 4.84
12	MC-LR & -LA	80.06 ± 16.26	77.09 ± 16.48	87.33 ± 4.77
13	MC-LR	15.18 ± 2.64	17.40 ± 4.55	17.07 ± 4.32
14	MC-LR & -LA	137.19 ± 25.79	147.51 ± 21.27	91.46 ± 26.56

a.

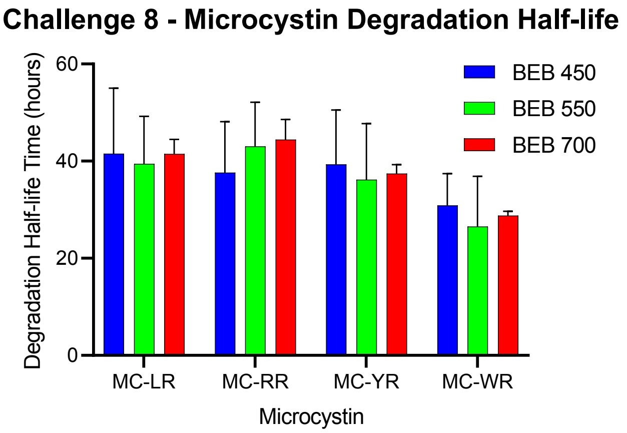

Microcystin	Incubation Time Required for 50 % Microcystin Degradation (hours)		
	BEB 450	BEB 550	BEB 700
MC-LR	41.55 ± 13.47	39.43 ± 9.81	41.49 ± 2.99
MC-RR	37.64 ± 10.49	43.02 ± 9.11	44.41 ± 9.11
MC-YR	39.30 ± 11.22	36.18 ± 11.54	37.43 ± 1.86
MC-WR	30.85 ± 6.57	26.51 ± 10.38	28.79 ± 0.90

b.

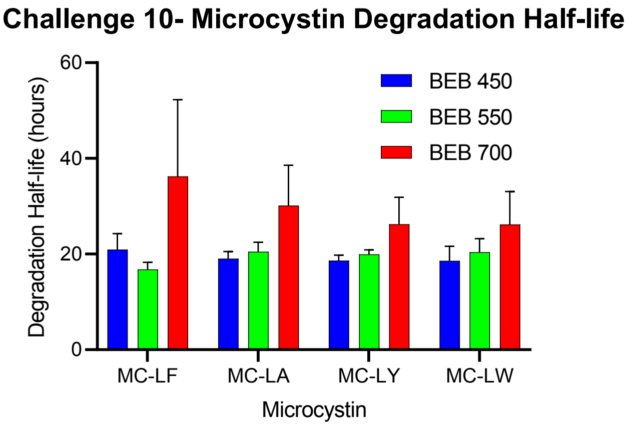

Microcystin	Incubation Time Required for 50 % Microcystin Degradation (hours)		
	BEB 450	BEB 550	BEB 700
MC-LF	20.93 ± 3.35	16.76 ± 1.53	36.23 ± 16.04
MC-LA	19.03 ± 1.50	20.48 ± 2.00	30.12 ± 8.48
MC-LY	18.60 ± 1.15	19.92 ± 0.98	26.23 ± 5.66
MC-LW	18.60 ± 3.04	20.37 ± 2.87	26.19 ± 6.86

Fig. S16 – BEB microcystin biodegradation half-life, challenge 8 and challenge 10.
The microcystin concentration was monitored by UPLC-PDA-MS/MS to assess the rate of biodegradation by the biologically enhanced coconut biochar. The time taken for 50 % of the each of microcystins to be degraded during **a.** challenge 8: MC-LR, -RR, -YR & -WR and **b.** challenge 10: MC-LF, -LA, -LY & -LW was then calculated. BEB 450, 550 & 700 refers to the HTT pyrolysis temperature on synthesis of the coconut shell biochar. Error bars represent the standard deviation n=3.

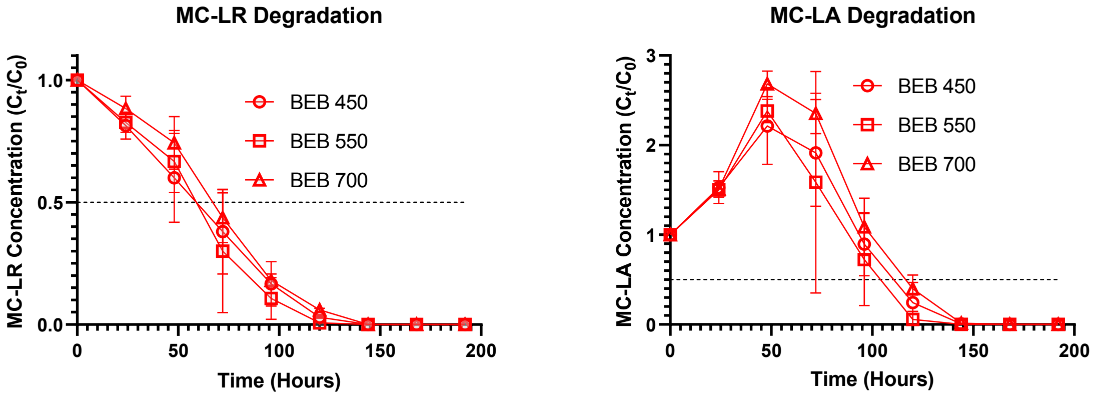

Fig. S17 - Assessing the ability of the BEB to degrade microcystins in with increased biological complexity, challenge 12: Cyanobacterial extract.

The biologically enhance coconut biochar was transferred into flasks containing sterile Rescobie Loch water and *Microcystis aeruginosa* B2666 cell extract. The cell extract contained MC-LR & -LA as well as other cellular components. The microcystin concentration was monitored over 192 hours by UPLC-PDA-MS/MS to assess the rate of MC-LR & -LA biodegradation by the BEBs. BEB 450, 550 & 700 refers to the HTT pyrolysis temperature on synthesis of the coconut shell biochar. Error bars represent the standard deviation n=3.

a.

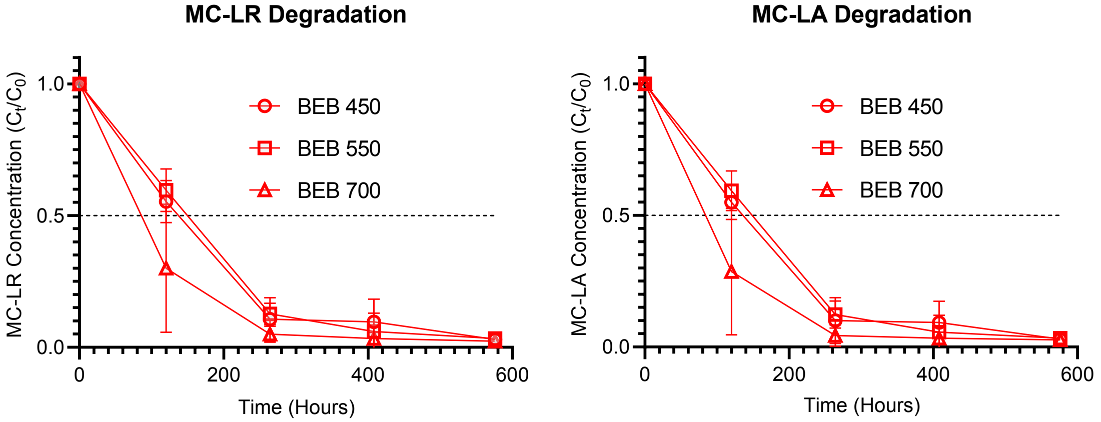

b.

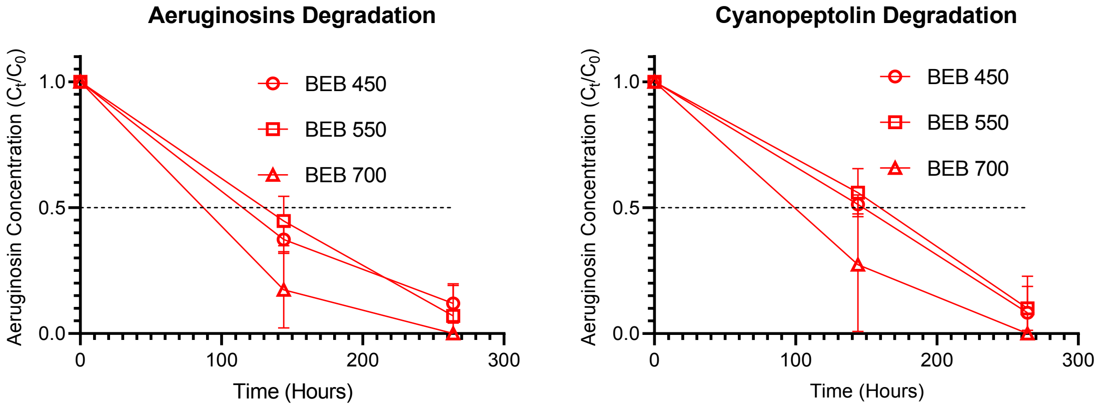

c.

**Toxin**	**Incubation Time Required for 50 % Microcystin Degradation (hours)**		
	**BEB 450**	**BEB 550**	**BEB 700**
MC-LR	137.89 ± 25.18	148.34 ± 21.03	91.93 ± 26.86
MC-LA	136.88 ± 21.63	147.85 ± 21.04	89.81 ± 25.44
Aeruginosins	115.46 ± 9.68	133.57 ± 26.92	88.91 ± 14.88
Cyanopeptolin	148.53 ± 11.16	159.27 ± 25.35	108.07 ± 39.82

Fig. S18 – BEB Cyanotoxin degradation in the presence of live cyanobacteria, challenge 14. The BEBs were transferred into flasks containing sterile Rescobie Loch water and cyanotoxin producing live *Microcystis aeruginosa* B2666 cells. These cells were producing MC-LR & -LA, as well as aeruginosins and cyanopeptolin. During challenge 14, **a.** the MC-LR & -LA concentration was monitored by UPLC-PDA-MS/MS and **b.** the aeruginosins & cyanopeptolin concentration by UPLC–PDA–QTOF–MS^E and –MS/MS. **c.** The time taken for 50 % of each toxin produced by live cyanobacteria (MC-LR, -LA, aeruginosins & cyanopeptolin) to be degraded was calculated. BEB 450, 550 & 700 refers to the HTT pyrolysis temperature on synthesis of the coconut shell biochar. Error bars represent the standard deviation n=3.
